# PD Control with Feedforward Compensation for String Stable Cooperative Adaptive Cruise Control in Vehicle Platoons

**DOI:** 10.3390/s25175434

**Published:** 2025-09-02

**Authors:** Kangjun Lee, Chanhwa Lee

**Affiliations:** 1 Department of Artificial Intelligence and Robotics, Sejong University, Seoul 05006, Republic of Korea; kangjun.lee@sju.ac.kr; 2 Artificial Intelligence and Robotics Institute, Sejong University, Seoul 05006, Republic of Korea

**Keywords:** CACC, string stability, platoon, PD control, feedforward compensation

## Abstract

In this paper, we propose systematic controller design guidelines to ensure both individual vehicle stability and string stability in cooperative adaptive cruise control (CACC)-based platoon systems, assuming a homogeneous platoon where all vehicles share identical dynamic models. We rigorously demonstrate that the limitation of conventional adaptive cruise control (ACC) in maintaining the target inter-vehicle distance can be effectively overcome by incorporating the desired acceleration of the preceding vehicle as a static feedforward input. Furthermore, by formulating transfer functions in the frequency domain, we analytically derive the conditions required to ensure both individual vehicle stability and string stability of the CACC system. Building on this insight, we propose a practical and theoretically well-founded design guideline for determining the proportional, derivative, and feedforward gains of control input under a constant time gap spacing policy. The proposed guidelines are validated through simulations conducted in a realistic platooning scenario involving multiple vehicles.

## 1. Introduction

This paper extends our recent work on the rigorous analysis and systematic design of conventional adaptive cruise control (ACC) systems for string stability [1] by focusing on cooperative ACC (CACC). CACC leverages vehicle-to-vehicle (V2V) communication to access the desired acceleration of the preceding vehicle (i.e., $u_{i-1}:=a_{d,i-1}$), thereby enabling significantly shorter inter-vehicle distances compared to conventional ACC. As such, CACC is considered a key enabler of vehicle platooning, where multiple vehicles travel in a closely coordinated formation. While conventional ACC relies solely on on-board sensors, such as radar, LiDAR, and cameras, detecting surrounding vehicles to maintain relatively long following distances for safety and ride comfort [2,3], our previous study highlighted the role of ACC as a baseline platooning controller when communication for CACC is unavailable, and proposed a corresponding controller design method. The main distinction of the present work lies in the inclusion of a static feedforward term in the control structure, which utilizes the desired acceleration of the preceding vehicle. Although this modification may appear relatively minor, it significantly complicates the analytical framework required to guarantee stability properties. This paper rigorously addresses these analytical challenges and derives new stability conditions tailored to the CACC setting. We fully acknowledge that the benefits of feedforward control in improving string stability—particularly by allowing for a smaller required headway distance—have been well established in the literature. This is because feedback-only control is fundamentally constrained by the so-called waterbed effect, a consequence of the Bode sensitivity integral. In this context, our analysis not only confirms the well-known theoretical advantage of feedforward structures but also quantitatively demonstrates that the minimum achievable headway distance can be significantly reduced compared to conventional ACC.

Such improvement of CACC directly contributes to increased traffic efficiency, enhanced safety, and economic benefits, thereby highlighting the practical value of the proposed CACC-based platooning design. First, cooperative control among platooning vehicles increases road capacity and improves travel time reliability, particularly in high-density traffic environments. In terms of traffic management, CACC also mitigates congestion by smoothing traffic flow and reducing stop-and-go behavior. Another major advantage of CACC lies in its ability to significantly enhance road safety. By enabling real-time V2V communication and coordinated control, CACC reduces reliance on human judgment, allowing vehicles to maintain shorter following distances and respond more rapidly to dynamic traffic conditions. This helps prevent accidents commonly caused by delayed human reactions and inattentive driving [4,5,6]. An even more significant benefit of CACC lies in its economic impact on freight transportation, where truck platooning substantially reduces fuel consumption and operational costs. Truck platooning notably reduces aerodynamic drag for following vehicles, leading to improved fuel efficiency and reduced operating costs [7,8,9,10,11,12]. Furthermore, smoother acceleration and braking reduce wear on mechanical components and lower emissions, aligning with both cost-saving and environmental objectives.

String stability [13,14], which refers to the property that disturbances are attenuated rather than amplified as they propagate through a vehicle platoon, is a critical requirement for ensuring safety and maintaining smooth traffic flow. Extensive research has been conducted on the development and analysis of string stability in both ACC and CACC systems. Studies on ACC-based platoons have shown that string stability can be achieved under specific control designs and spacing policies, but their limited ability to respond to upstream vehicle behavior necessitates relatively long inter-vehicle distances [15,16]. In contrast, CACC enhances the vehicle following performance by incorporating V2V communication. This cooperative control framework significantly improves responsiveness and coordination, enabling vehicles to maintain shorter and more stable inter-vehicle distances without compromising safety [17,18,19,20]. As a result, CACC not only achieves superior string stability compared to ACC but also supports tighter platooning formations that are essential for improving traffic throughput and fuel efficiency in automated highway systems.

### 1.1. Contributions

Ensuring string stability is essential for the practical deployment and commercialization of CACC, and thus, a critical challenge lies in developing controller designs that not only have a solid theoretical foundation but are also feasible for real-world implementation. This paper presents a rigorous analysis and a systematic design methodology for CACC-based platoon systems with homogeneous vehicle dynamics operating under the constant time gap (CTG) policy. Despite the extensive literature on CACC, existing studies often rely on either idealized assumptions or complex controller structures without providing systematic guidelines for selecting controller parameters that ensure both individual vehicle stability and string stability. The main contributions of this paper are summarized as follows:

(1) We propose a practical CACC controller structure based on proportional-derivative (PD) feedback combined with static feedforward compensation. This design offers ease of implementation while maintaining strong performance guarantees. To address the platoon control problem, various approaches have been proposed in the literature, including sliding mode control [21,22], $H_{\infty }$ control [23,24], event-triggered control [25], adaptive optimal control [26], and, more recently, reinforcement learning approaches [27,28]. In contrast, the proposed method adopts a significantly simpler structure, consisting of a PD feedback controller and a static feedforward term, offering a more straightforward and practically implementable alternative to many existing approaches. Specifically, while the feedback loop is realized using a PD controller, the feedforward component applies a tunable static gain to the desired acceleration of the preceding vehicle, in contrast to prior studies that typically employ fixed-parameter dynamic compensators based on filtered actual acceleration signals, as seen in [29,30].
(2) The results in [29,30,31] may appear similar to ours, as their feedback controllers are variations of PD control and their feedforward components also employ V2V communication information. However, in the aforementioned studies, including those not based on PD control, the controllers require the actual longitudinal acceleration, either in the feedback or in the feedforward loop. This actual acceleration can be distorted by road grade when measured via accelerometers, or by tire slip when estimated through the differentiation of wheel speed. Consequently, a wide range of techniques have been proposed to address the critical challenge of accurately estimating acceleration. For example, some approaches employ Gaussian-process-based model predictive control [32] or neural networks with long short-term memory (LSTM) architectures [33], while others, such as [34], propose sliding mode estimation techniques. Moreover, to address situations where the acceleration of the preceding vehicle is unavailable, degraded-CACC strategies have been developed, which remove the communication component by estimating the acceleration of the preceding vehicle via backward derivative approximation [35]. In contrast, our approach eliminates the need for actual longitudinal acceleration in both the feedback and feedforward loops, relying solely on desired acceleration in the feedforward path, thereby offering a potentially novel and practical implementation.
(3) We derive necessary and sufficient conditions for both individual vehicle stability and string stability. By adopting the frequency-domain analysis framework proposed in [36], we obtain explicit analytical ranges for the controller gains through a rigorous derivation. In contrast, Ref. [36] determines the minimum time gap constant numerically and assumes ideal actuation dynamics, which limits the generality and practical applicability of its results. Furthermore, the conditions for individual vehicle stability are also explicitly formulated as bounds on the controller gains.
(4) We provide a clear and systematic design guideline based on an analytical characterization of the feasible parameter regions. This guideline enables practitioners to select controller parameters in a principled manner to ensure both stability and desired performance.
(5) We also develop practical design strategies that explicitly account for communication delays, which are inherent in real-world vehicle networks and can degrade stability and performance if left unaddressed. Additionally, we examine a CACC variant that utilizes the *actual* acceleration of the preceding vehicle in the feedforward term, which is relevant in scenarios where the *desired* acceleration is unavailable, such as when the preceding vehicle is manually driven. This analysis clarifies that the use of the desired acceleration in the proposed control law allows for a smaller time gap, thereby improving traffic efficiency without compromising string stability.

### 1.2. Organization

The remainder of this paper is organized as follows. Section 2 introduces the CACC control framework and formally defines the problem. Section 3 derives the conditions required for both individual vehicle stability and string stability. Section 4 presents the main results that establish the design guidelines for the PD and feedforward controller, and Section 5 validates the proposed method through simulation studies. Finally, Section 6 concludes the paper and outlines future research directions, including extensions to uncertain heterogeneous vehicle platoons.

## 2. Problem Formulations

This section presents the formulation of the CACC problem considered in this study. We start by modeling the longitudinal dynamics of each vehicle and stating the assumptions that guide the control design process. Next, we define essential concepts, including individual vehicle stability and string stability. We then describe the desired behavior of the vehicle platoon and outline the corresponding control objectives. These formulations provide the foundation for the controller design presented in the following sections.

### 2.1. CACC Under Constant Time Gap Policy

**Figure 1:**
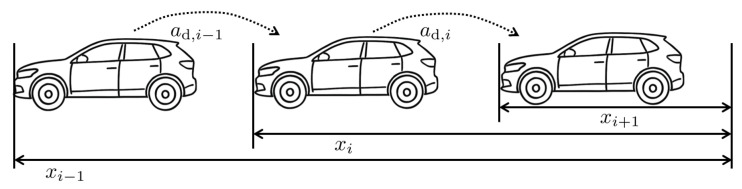
Vehicle platoon equipped with V2V communication.

As depicted in Figure 1, vehicle platooning refers to a group of vehicles traveling together while maintaining short distances between them by using information shared through V2V communication. To achieve this, CACC controllers have been widely adopted, which enhance conventional ACC by leveraging V2V communication (e.g., the desired acceleration $a_{d,i}$ in Figure 1) to improve spacing control and stability. In CACC, the CTG spacing policy, in which the desired inter-vehicle spacing is determined by multiplying the velocity $v_{i}:={\dot{x}}_{i}$ of the following vehicle by the constant time gap *h*, is commonly employed [37]. Specifically, for vehicle index *i*, if the actual spacing between the (i−1)-th and *i*-th vehicles is defined as $d_{i}:=x_{i-1}-x_{i}$, then the desired spacing $d_{d,i}$ is given by

$$\begin{array}{c} d_{d,i}:=hv_{i}. \end{array}$$

Consequently, the control objective is to regulate the actual spacing $d_{i}$ so that it tracks the desired spacing $d_{d,i}$. This can be formulated as minimizing the spacing error given by

(1) $$\begin{array}{c} e_{x,i}\left(t\right):=d_{i}-d_{d,i}=x_{i-1}\left(t\right)-x_{i}\left(t\right)-hv_{i}\left(t\right), \end{array}$$

and the goal is to ensure that $e_{x,i}\left(t\right)$ asymptotically converges to zero. To fully exploit the benefits of a CACC-based platoon with short inter-vehicle distances, a smaller value of *h* is preferred, and hence, the time gap *h* is typically chosen to be less than one. In the following section, we present a systematic controller design method that ensures stability for a given desired time gap *h*.

### 2.2. Homogeneous Platoon and Longitudinal Vehicle Dynamics

**Figure 2:**
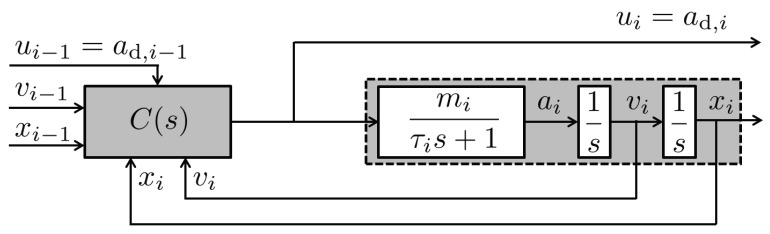
Controller structure and longitudinal vehicle dynamics.

The CACC-based control scheme and longitudinal vehicle dynamics are summarized in Figure 2. Let the subscript *i* represent the index of each vehicle in the platoon. The vehicle’s acceleration dynamics, mapping the desired acceleration input $a_{d,i}$ to the actual acceleration output $a_{i}$, are modeled using a first-order system with time constant $\tau_{i}$ and a DC gain $m_{i}$. Consequently, by treating the desired acceleration $a_{d,i}$ as the control input $u_{i}$ and the vehicle position $x_{i}$ as the output $y_{i}$, the longitudinal behavior of each vehicle can be modeled as the following third-order system:

(2) $$\begin{array}{c} P_{i}\left(s\right)=\frac{Y_{i}\left(s\right)}{U_{i}\left(s\right)}=\frac{X_{i}\left(s\right)}{A_{d,i}\left(s\right)}=\frac{1}{s^{2}}\frac{A_{i}\left(s\right)}{A_{d,i}\left(s\right)}=\frac{m_{i}}{s^{2}\left(\tau_{i}s+1\right)}, \end{array}$$

where capital letters represent the Laplace transforms of the respective time-domain signals. Here, $\tau_{i}>0$ captures the actuation delay introduced by the vehicle’s powertrain and mechanical response, whereas $m_{i}>0$ accounts for differences in vehicle mass, such as those arising from load variations or model uncertainties. Although the longitudinal model in (2) is relatively simple, it effectively represents the core dynamics of a vehicle’s longitudinal behavior. Hence, it has been widely adopted in numerous studies on longitudinal vehicle control [1,22,23,25,29,30,31,36,37,38]. While many previous studies often adopt the assumption $m_{i}=1$ for simplicity [22,23,25,29,30,31,37,38], this work treats $m_{i}$ as a variable parameter that may deviate from unity.

In this paper, we adopt a modeling assumption of a homogeneous platoon, where the longitudinal dynamics of all vehicles are the same, that is, $m_{i}=m$ and $\tau_{i}=\tau$ for all *i*. This assumption is formally stated as follows:

**Assumption** **1.** *All vehicles in the platoon are assumed to have identical longitudinal dynamics. That is, the transfer function (2) of the i-th vehicle is given by*

(3) $$\begin{array}{c} P_{i}\left(s\right)=P\left(s\right)=\frac{m}{s^{2}\left(\tau s+1\right)}\;\;\mathrm{for}\;\mathrm{all}\;\;i, \end{array}$$

*where τ>0 is the time constant representing the drivetrain delay, and m>0 denotes the DC gain.*

### 2.3. Stability in Platoon: Individual Vehicle Stability and String Stability

In a platoon system, each vehicle must maintain a desired spacing from the one in front. To guarantee both safety and efficiency, the control system must satisfy two key performance criteria, namely, individual vehicle stability and string stability [25,37]. First, individual vehicle stability refers to a vehicle’s ability to reduce its spacing error to zero, typically when the preceding vehicle drives at a constant velocity. The definition of individual vehicle stability adopted in this paper is outlined as follows.

**Definition** **1** ([25], Section III.B)**.** *A platoon system is said to be individually vehicle stable if, for each vehicle i, the spacing error $e_{x,i}\left(t\right)$ in *(1)* satisfies*

$$\lim_{t\to \infty }e_{x,i}\left(t\right)=0,$$

*provided that the acceleration and control input of the preceding vehicle remain zero, i.e., ${\ddot{x}}_{i-1}\left(t\right)=0$ and $u_{i-1}\left(t\right)=0$ for all t>0.*

Based on the above definition, the following lemma provides a condition for verifying individual vehicle stability using the system’s transfer function.

**Lemma** **1.** *The i-th vehicle in a platoon satisfies individual stability if and only if the transfer function from the preceding vehicle’s acceleration $a_{i-1}={\ddot{x}}_{i-1}$ to the spacing error $e_{x,i}$, defined as*

(4) $$\begin{array}{c} G_{i}\left(s\right):=\frac{E_{x,i}\left(s\right)}{A_{i-1}\left(s\right)}=\frac{E_{x,i}\left(s\right)}{s^{2}X_{i-1}\left(s\right)}, \end{array}$$

*is stable; that is, all poles of $G_{i}\left(s\right)$ have strictly negative real parts.*

**Proof.**  Assume that $u_{i-1}\left(t\right)=0$ for all t≥0, and consider the corresponding linear time-invariant (LTI) system with input $a_{i-1}\left(t\right)={\ddot{x}}_{i-1}\left(t\right)$ and output $e_{x,i}\left(t\right)$. Then, $G_{i}\left(s\right)$ in (4) denotes the transfer function from the input $a_{i-1}\left(t\right)$ to the output $e_{x,i}\left(t\right)$. According to standard results in linear systems theory, the zero-input response of an LTI system converges to zero as t→∞ if and only if all poles of the transfer function $G_{i}\left(s\right)$ lie strictly in the open left-half complex plane, i.e., $G_{i}\left(s\right)$ is a stable transfer function. Since the zero input response corresponds to the case where $a_{i-1}\left(t\right)=0$ for all t≥0, it follows that the system is individually vehicle stable if and only if $G_{i}\left(s\right)$ is stable. This completes the proof.    □

Although individual vehicle stability guarantees that each vehicle can correct its own spacing error, it does not necessarily imply the overall stability for a CACC-based platoon system. When the lead vehicle changes its velocity, tracking errors are generated and passed down to the following vehicles. As a result, even if each vehicle is individually stable on its own, the accumulated effect of these errors can cause oscillatory behavior or even lead to inter-vehicle collisions in extreme cases. To address this issue, the notion of string stability is introduced [13,14]. String stability ensures that disturbances, such as sudden acceleration or braking, do not grow as they propagate along the vehicle string, thereby playing a crucial role in reducing traffic fluctuations and enhancing both safety and throughput in platoon operations.

String stability is a formal condition that ensures the spacing error $e_{x,i}$, as defined in (1), does not grow as it propagates through the vehicle string. This condition is typically characterized by the inequality $∥e_{x,i}∥\leq ∥e_{x,i-1}∥$, which can also be written as follows:

(5) $$\begin{array}{c} ∥\frac{e_{x,i}}{e_{x,i-1}}∥\leq 1. \end{array}$$

Let the transfer function that describes the propagation of spacing errors from vehicle (i−1) to vehicle *i* be defined as the ratio of the Laplace transform of $e_{x,i}\left(t\right)$ to that of $e_{x,i-1}\left(t\right)$,

(6) $$\begin{array}{c} H_{i}\left(s\right):=\frac{E_{x,i}\left(s\right)}{E_{x,i-1}\left(s\right)}. \end{array}$$

The condition in (5) to $H_{i}\left(s\right)$ defines the concept of the frequency-domain string stability as follows.

**Definition** **2** ([14], Section 4.2)**.** *A platoon system is said to be string stable if, for each vehicle i, the transfer function of spacing errors between the (i−1)-th vehicle and i-th vehicle, denoted as $H_{i}\left(s\right)$ in (6), satisfies*

$$\begin{array}{c} {∥H_{i}\left(j\omega \right)∥}_{\infty }\leq 1, \end{array}$$

*where ${∥H_{i}\left(j\omega \right)∥}_{\infty }$ denotes the $H_{\infty }$ norm of $H_{i}\left(j\omega \right)$, the maximum magnitude of the transfer function.*

Definition 2 can be equivalently expressed as a frequency-domain condition, requiring that the magnitude of the transfer function does not exceed unity across all frequencies. The following lemma provides a formal statement of this condition.

**Lemma** **2.** *A platoon system is string stable if and only if the Bode magnitude plot of the error-propagation transfer function $H_{i}\left(s\right)$, defined in (6), remains less than or equal to one for all frequencies:*

(7) $$\begin{array}{c} |H_{i}\left(j\omega \right)|\leq 1,\;\forall \omega \geq 0. \end{array}$$

**Proof.**  The proof is straightforward by the definition of the $H_{\infty }$ norm.    □

### 2.4. Control Objectives with PD Controller and Feedforward Compensator

Consider a homogeneous vehicle platoon where each vehicle follows identical longitudinal dynamics modeled by (3). The desired inter-vehicle spacing is defined by the CTG policy $d_{d,i}\left(t\right)=hv_{i}\left(t\right)$, where h>0 is a constant time gap. Each vehicle can access its own velocity, the relative distance and velocity with respect to the preceding vehicle, and the desired acceleration of the preceding vehicle via V2V communication. The control objective is to ensure that the actual spacing $d_{i}\left(t\right)$ accurately tracks the desired spacing $d_{d,i}\left(t\right)=h{\dot{x}}_{i}\left(t\right)$, even under realistic and potentially time-varying acceleration profiles of the lead vehicle. To achieve this, the control input for the *i*-th vehicle can be designed using a PD controller with a feedforward term, given by

(8) $$\begin{array}{c} u_{i}\left(t\right)=a_{d,i}\left(t\right)=k_{FF}a_{d,i-1}\left(t\right)+k_{p}\left(x_{i-1}\left(t\right)-x_{i}\left(t\right)-hv_{i}\left(t\right)\right)+k_{d}\left(v_{i-1}\left(t\right)-v_{i}\left(t\right)\right), \end{array}$$

where $k_{FF}$ is the static feedforward gain associated with the desired acceleration from the preceding vehicle, $k_{p}$ is the proportional gain for relative distance and its own velocity, and $k_{d}$ is the derivative gain for relative velocity. If $k_{FF}=0$, the controller reduces to a conventional ACC system that relies solely on feedback measurements. If $k_{FF}\neq 0$, the controller becomes a CACC system that utilizes feedforward information from the preceding vehicle via V2V communication, enabling improved tracking performance and string stability.

The feedforward control in (8) utilizes the desired acceleration, in contrast to previous works [29,30,36], which employ the actual acceleration of the preceding vehicle in conjunction with a dynamic feedforward filter. Although the use of actual acceleration with a dynamic filter facilitates system analysis, the accurate measurement of longitudinal acceleration is often hindered by factors such as tire slip and road grade. For instance, acceleration measured using accelerometers can be distorted by road slope, whereas estimation based on the differentiation of wheel speed is susceptible to errors caused by tire slip. In contrast, the proposed control law (8) presents a practical alternative by using the desired acceleration as the feedforward signal, offering a novel approach that avoids the need to measure potentially inaccurate actual acceleration. Note that the leading vehicle may be manually driven by a human. In such cases, the desired acceleration of the preceding vehicle for the second vehicle, i.e., the first following vehicle behind the leading vehicle, is not directly available. A practical alternative is to preprocess the actual acceleration signal using brake pedal position data, as suggested in [39]. In contrast, if the leading vehicle is operated by an advanced driver assistance system (ADAS), its desired acceleration can be directly utilized as the feedforward input for the second vehicle.

Finally, the control design problem is to determine the gains $k_{FF}$, $k_{p}$, and $k_{d}$ such that the following objectives are satisfied:

**Problem** **1** (PD-Based CACC Design under the CTG Policy)**.** *Given a homogeneous platoon of vehicles with longitudinal dynamics described by *(3)*, design a PD controller with a feedforward compensator of the form *(8)* that ensures both individual vehicle stability (Definition 1) and string stability (Definition 2) under the CTG policy defined in *(1)*.*

## 3. Stability Analysis on Individual Vehicle Stability and String Stability

In our previous study [1], we provided controller design guidelines for ACC, which is fundamentally limited by the condition h>2τ to satisfy string stability. In other words, ACC requires a predefined minimum headway distance of $2\tau v_{i}$ to maintain stability. In this section, we rigorously analyze how CACC can overcome this limitation and achieve smaller desired inter-vehicle spacing. Based on this analysis, we derive the conditions that the control gains must satisfy to ensure both individual vehicle stability and string stability. For the reader’s convenience, Table 1 summarizes the stability conditions along with their corresponding transfer functions and parameter-based expressions. The detailed derivations of each condition are presented in Section 3.1, Section 3.2 and Section 3.3. Additionally, Section 3.4 addresses the impact of communication delay and presents controller design strategies to ensure stability and performance in its presence. Lastly, the feedforward term is slightly modified in Section 3.5 to incorporate the *actual* acceleration instead of the *desired* one. These variations are considered to clarify the rationale for adopting the desired acceleration in the final control law (8).

### 3.1. Transfer Function Representations and Frequency-Domain Analysis

Let us start with applying the Laplace transform to the control input designed in (8), which yields the following:

(9) $$\begin{array}{c} \;\begin{array}{cc} U_{i}\left(s\right) & =k_{FF}U_{i-1}\left(s\right)+k_{p}\left(X_{i-1}\left(s\right)-X_{i}\left(s\right)-hsX_{i}\left(s\right)\right)+k_{d}\left(sX_{i-1}\left(s\right)-sX_{i}\left(s\right)\right)\\ \; & =k_{FF}U_{i-1}\left(s\right)+k_{p}P\left(s\right)\left(U_{i-1}\left(s\right)-U_{i}\left(s\right)-hsU_{i}\left(s\right)\right)+k_{d}sP\left(s\right)\left(U_{i-1}\left(s\right)-U_{i}\left(s\right)\right).\;\;\;\;\; \end{array} \end{array}$$

Under the assumption of identical vehicle dynamics within the platoon, by rearranging (9), we derive the transfer function from $U_{i-1}\left(s\right)$ to $U_{i}\left(s\right)$, which remains independent of the vehicle index *i* as follows:

(10) $$\begin{array}{c} \begin{array}{cc} \Gamma_{i}\left(s\right):=\frac{U_{i}\left(s\right)}{U_{i-1}\left(s\right)} & =\frac{k_{FF}+P\left(s\right)\left(k_{d}s+k_{p}\right)}{1+P\left(s\right)\left(hk_{p}s+k_{d}s+k_{p}\right)}\\ \; & =\frac{k_{FF}s^{2}\left(\tau s+1\right)+m\left(k_{d}s+k_{p}\right)}{s^{2}\left(\tau s+1\right)+m\left(hk_{p}s+k_{d}s+k_{p}\right)}\\ \; & =\frac{\tau k_{FF}s^{3}+k_{FF}s^{2}+mk_{d}s+mk_{p}}{\tau s^{3}+s^{2}+m\left(hk_{p}+k_{d}\right)s+mk_{p}}=:\Gamma \left(s\right). \end{array} \end{array}$$

Using (10), the transfer function $G_{i}\left(s\right)$ in (4), which serves to evaluate individual vehicle stability, can be expressed as follows (importantly, it does not depend on the vehicle index *i*):

(11) $$\begin{array}{c} \begin{array}{cc} G_{i}\left(s\right):= & \frac{E_{x,i}\left(s\right)}{A_{i-1}\left(s\right)}=\frac{E_{x,i}\left(s\right)}{X_{i-1}\left(s\right)}\frac{1}{s^{2}}\\ \; & =\frac{X_{i-1}\left(s\right)-X_{i}\left(s\right)-hsX_{i}\left(s\right)}{X_{i-1}\left(s\right)}\frac{1}{s^{2}}\\ \; & =\frac{P\left(s\right)\left(U_{i-1}\left(s\right)-U_{i}\left(s\right)-hsU_{i}\left(s\right)\right)}{P\left(s\right)U_{i-1}\left(s\right)}\frac{1}{s^{2}}=\left(1-(hs+1)\Gamma (s)\right)\frac{1}{s^{2}}\\ \; & =\frac{-h\tau k_{FF}s^{2}+\left(\tau \left(1-k_{FF}\right)-hk_{FF}\right)s+\left(\left(1-k_{FF}\right)-mhk_{d}\right)}{\tau s^{3}+s^{2}+m\left(hk_{p}+k_{d}\right)s+mk_{p}}=:G\left(s\right). \end{array} \end{array}$$

Based on Lemma 1, the transfer function G(s) must be stable to ensure individual vehicle stability, that is, its characteristic polynomial must be Hurwitz, meaning all poles lie strictly in the open left half of the complex plane. Accordingly, we define the characteristic polynomial D(s), which is shared by both Γ(s) and G(s), as

(12) $$\begin{array}{c} D\left(s\right):=\tau s^{3}+s^{2}+m\left(hk_{p}+k_{d}\right)s+mk_{p}. \end{array}$$

If D(s) is Hurwitz, then G(s) is stable, and the platoon system satisfies individual vehicle stability as defined in Definition 1.

Now, to analyze string stability, we derive the transfer function $H_{i}\left(s\right)$ in (6) as follows:

(13) $$\begin{array}{c} \begin{array}{cc} H_{i}\left(s\right) & :=\frac{E_{x,i}\left(s\right)}{E_{x,i-1}\left(s\right)}=\frac{X_{i-1}\left(s\right)-X_{i}\left(s\right)-hsX_{i}\left(s\right)}{X_{i-2}\left(s\right)-X_{i-1}\left(s\right)-hsX_{i-1}\left(s\right)}\\ \; & =\frac{P\left(s\right)\left(U_{i-1}\left(s\right)-U_{i}\left(s\right)-hsU_{i}\left(s\right)\right)}{P\left(s\right)\left(U_{i-2}\left(s\right)-U_{i-1}\left(s\right)-hsU_{i-1}\left(s\right)\right)}\\ \; & =\frac{\Gamma \left(s\right)\left(U_{i-2}\left(s\right)-U_{i-1}\left(s\right)-hsU_{i-1}\left(s\right)\right)}{U_{i-2}\left(s\right)-U_{i-1}\left(s\right)-hsU_{i-1}\left(s\right)}=\Gamma \left(s\right)=:H\left(s\right). \end{array} \end{array}$$

Note that it is independent of the vehicle index *i*, and coincides with the transfer function Γ(s) defined in (10). From (13), string stability condition (7) in Lemma 2 changes to

(14) $$\begin{array}{c} |\Gamma (j\omega )|\leq 1,\;\forall \omega \geq 0. \end{array}$$

If the above condition (14) holds, the platoon system achieves string stability. It is worth noting that if H(s) is defined as the error string stability function and Γ(s) as the input string stability function, then they are identical for a homogeneous platoon. This observation is consistent with the result reported in [36] and will be further validated through simulations in Section 5.

### 3.2. Individual Vehicle Stability for CACC

In the remaining subsections, we present a detailed and rigorous analysis of the conditions that ensure both individual stability and string stability. First, to ensure individual stability, D(s) in (12) must be Hurwitz. By applying Routh’s stability criterion ([40], Chapter 3.6) to D(s), we derive the conditions to make D(s) Hurwitz. According to Routh’s stability criterion, a transfer function is Hurwitz stable if there are no sign changes in the first column of its Routh array. The Routh array of D(s), computed as shown in Table 2, leads to the following conditions that must be satisfied to ensure that all elements in the first column remain positive:

(15a) $$\begin{array}{cc} k_{p} & >0, \end{array}$$

(15b) $$\begin{array}{cc} k_{d} & >\left(\tau -h\right)k_{p}. \end{array}$$

Satisfaction of the conditions in (15) ensures individual vehicle stability for the platoon system.

### 3.3. String Stability for CACC (with Feedforward Control Using the Desired Acceleration)

When $k_{FF}=0$, the system reduces to a conventional ACC controller, which has been thoroughly analyzed in [1]. Accordingly, we focus on the case $k_{FF}\neq 0$, corresponding to CACC, and use the transfer function Γ(s) in (10) to analyze string stability.

$$\begin{array}{c} \Gamma \left(s\right)=\frac{\tau k_{FF}s^{3}+k_{FF}s^{2}+mk_{d}s+mk_{p}}{\tau s^{3}+s^{2}+m\left(hk_{p}+k_{d}\right)s+mk_{p}}. \end{array}$$

To guarantee string stability, the magnitude of Γ(jω) must hold

(16) $$\begin{array}{c} {\left|\Gamma \left(j\omega \right)\right|}^{2}=\frac{{\left(mk_{p}-k_{FF}\omega^{2}\right)}^{2}+{\left(mk_{d}\omega -\tau k_{FF}\omega^{3}\right)}^{2}}{{\left(mk_{p}-\omega^{2}\right)}^{2}+{\left(m\left(hk_{p}+k_{d}\right)\omega -\tau \omega^{3}\right)}^{2}}\leq 1,\;\forall \omega \geq 0, \end{array}$$

from the condition (14). This inequality is equivalently expressed as

$$\begin{array}{cc} \left({\left(mk_{p}-\omega^{2}\right)}^{2}+{\left(m\left(hk_{p}+k_{d}\right)\omega -\tau \omega^{3}\right)}^{2}\right) & \\ -\left({\left(mk_{p}-k_{FF}\omega^{2}\right)}^{2}+{\left(mk_{d}\omega -\tau k_{FF}\omega^{3}\right)}^{2}\right) & \geq 0,\;\forall \omega \geq 0,\\ \tau^{2}\left(1-k_{FF}^{2}\right)\omega^{6}+\left(\left(1-k_{FF}^{2}\right)-2m\tau \left(hk_{p}+\left(1-k_{FF}\right)k_{d}\right)\right)\omega^{4} & \;\\ +\left(m^{2}{\left(hk_{p}+k_{d}\right)}^{2}-2m\left(1-k_{FF}\right)k_{p}-m^{2}k_{d}^{2}\right)\omega^{2} & \geq 0,\;\forall \omega \geq 0. \end{array}$$

By introducing the substitution $\chi :=\omega^{2}$, the inequality can be reformulated into the following quadratic form:

$$\begin{array}{cc} \tau^{2}\left(1-k_{FF}^{2}\right)\chi^{2}+\left(\left(1-k_{FF}^{2}\right)-2m\tau \left(hk_{p}+\left(1-k_{FF}\right)k_{d}\right)\right)\chi & \\ +\left(m^{2}{\left(hk_{p}+k_{d}\right)}^{2}-2m\left(1-k_{FF}\right)k_{p}-m^{2}k_{d}^{2}\right) & \geq 0,\;\forall \chi \geq 0. \end{array}$$

For notational simplicity, let us define the coefficients

(17) $$\begin{array}{c} \begin{array}{cc} \; & a:=\tau^{2}\left(1-k_{FF}^{2}\right),\\ \; & b:=\left(1-k_{FF}^{2}\right)-2m\tau \left(hk_{p}+\left(1-k_{FF}\right)k_{d}\right),\\ \; & c:=m^{2}{\left(hk_{p}+k_{d}\right)}^{2}-2m\left(1-k_{FF}\right)k_{p}-m^{2}k_{d}^{2}, \end{array} \end{array}$$

and the discriminant of the quadratic equation

$$\Delta :=b^{2}-4ac.$$

Consider the condition under which the quadratic function

(18) $$\begin{array}{c} f\left(\chi \right)=a\chi^{2}+b\chi +c\geq 0\;\mathrm{for}\;\mathrm{all}\;\chi \geq 0, \end{array}$$

is satisfied. To ensure that (18) holds over the non-negative real line, it is required that the leading coefficient satisfy a≥0, which leads to the following bound on the feedforward gain:

$$\begin{array}{c} 1-k_{FF}^{2}\geq 0\;\left(\mathrm{or}\;-1\leq k_{FF}\leq 1\right). \end{array}$$

Before proceeding with the main analysis, we first establish that the strict inequality $a=\tau^{2}\left(1-k_{FF}^{2}\right)>0$ must hold when considering individual vehicle stability together with string stability. To see this, consider the two boundary cases. First, when $k_{FF}=1$, we obtain $b=-2mh\tau k_{p}<0$. By the individual vehicle stability condition (15a), this case violates the non-negativity requirement of f(χ) in (18). Second, when $k_{FF}=-1$, we have $b=-2m\tau (hk_{p}+2k_{d})$ and $c=m^{2}hk_{p}\left(hk_{p}+2k_{d}\right)-4mk_{p}$. In this case, imposing b≥0 requires $hk_{p}+2k_{d}\leq 0$, which leads to $c\leq -4mk_{p}<0$, again violating the condition (18). Hence, in light of both individual vehicle stability and string stability, the strict bound

(19) $$\begin{array}{c} 1-k_{FF}^{2}>0\;\left(\mathrm{or}\;\;-1<k_{FF}<1\right) \end{array}$$

must be imposed.

Now, given the strict inequality $a=\tau^{2}\left(1-k_{FF}^{2}\right)>0$, the CACC system is string stable if and only if one of the following conditions is satisfied:

(c1) (a>0 and) b≥0 and c≥0,
(c2) (a>0 and) b<0 and Δ≤0.

The condition (c1) can be calculated as follows:

(20) $$\begin{array}{cccccc} \; & \left(1-k_{FF}^{2}\right)-2m\tau \left(hk_{p}+\left(1-k_{FF}\right)k_{d}\right)\geq 0\;\;\; & \; & \mathrm{and} & \; & \;\;\;m^{2}{\left(hk_{p}+k_{d}\right)}^{2}-2m\left(1-k_{FF}\right)k_{p}-m^{2}k_{d}^{2}\geq 0,\;\;\;\\ \; & hk_{p}+\left(1-k_{FF}\right)k_{d}\leq \frac{1}{2m\tau }\left(1-k_{FF}^{2}\right) & \; & \mathrm{and} & \; & \;\;\;m^{2}\left(h^{2}k_{p}^{2}+2hk_{p}k_{d}\right)-2m\left(1-k_{FF}\right)k_{p}\geq 0,\;\;\;\\ \; & hk_{p}+\left(1-k_{FF}\right)k_{d}\leq \frac{1}{2m\tau }\left(1-k_{FF}^{2}\right) & \; & \mathrm{and} & \; & \;\;\;hk_{p}+2k_{d}\geq \frac{2}{mh}\left(1-k_{FF}\right). \end{array}$$

By multiplying the second inequality in (20) by $\frac{1-k_{FF}}{2}$ and adding $\frac{1+k_{FF}}{2}hk_{p}$, the condition (20) can be equivalently expressed as requiring that the term $hk_{p}+\left(1-k_{FF}\right)k_{d}$ lies within the following bounds:

(21) $$\begin{array}{c} \frac{1}{mh}{\left(1-k_{FF}\right)}^{2}+\frac{1+k_{FF}}{2}hk_{p}\leq hk_{p}+\left(1-k_{FF}\right)k_{d}\leq \frac{1}{2m\tau }\left(1-k_{FF}^{2}\right). \end{array}$$

The condition (c2) can be expressed as follows:

(22) $$\begin{array}{c} \begin{array}{cc} \; & \left(1-k_{FF}^{2}\right)-2m\tau \left(hk_{p}+\left(1-k_{FF}\right)k_{d}\right)<0\;\mathrm{and}\\ \; & \;{\left(\left(1-k_{FF}^{2}\right)-2m\tau \left(hk_{p}+\left(1-k_{FF}\right)k_{d}\right)\right)}^{2}\\ \; & \;-4\tau^{2}\left(1-k_{FF}^{2}\right)\left(m^{2}{\left(hk_{p}+k_{d}\right)}^{2}-2m\left(1-k_{FF}\right)k_{p}-m^{2}k_{d}^{2}\right)\leq 0,\\ \; & hk_{p}+\left(1-k_{FF}\right)k_{d}>\frac{1}{2m\tau }\left(1-k_{FF}^{2}\right)\;\mathrm{and}\\ \; & \;{\left(\left(1-k_{FF}^{2}\right)-2m\tau \left(1-k_{FF}\right)k_{d}\right)}^{2}+4mh\tau k_{FF}k_{p}\left(\left(1-k_{FF}^{2}\right)-2m\tau \left(1-k_{FF}\right)k_{d}\right)\\ \; & \;+4m^{2}h^{2}\tau^{2}k_{FF}^{2}k_{p}^{2}-4mh\tau \left(1-k_{FF}^{2}\right)\left(1+k_{FF}\right)k_{p}+8m\tau^{2}\left(1-k_{FF}^{2}\right)\left(1-k_{FF}\right)k_{p}\leq 0,\\ \; & hk_{p}+\left(1-k_{FF}\right)k_{d}>\frac{1}{2m\tau }\left(1-k_{FF}^{2}\right)\;\mathrm{and}\\ \; & \;{\left(\left(\left(1-k_{FF}^{2}\right)-2m\tau \left(1-k_{FF}\right)k_{d}\right)+2mh\tau k_{FF}k_{p}\right)}^{2}\\ \; & \;\leq 4m\tau \left(1-k_{FF}^{2}\right)k_{p}\left(h\left(1+k_{FF}\right)-2\tau \left(1-k_{FF}\right)\right). \end{array} \end{array}$$

A simplified form of condition (22) is given by the following inequality:

(23) $$\begin{array}{c} \begin{array}{cc} \; & hk_{p}+\left(1-k_{FF}\right)k_{d}>\frac{1}{2m\tau }\left(1-k_{FF}^{2}\right)\;\mathrm{and}\\ \; & \;\;{\left(k_{d}-\left(\frac{1+k_{FF}}{2m\tau }+\frac{k_{FF}}{1-k_{FF}}hk_{p}\right)\right)}^{2}\leq \frac{k_{p}}{m\tau }\frac{{\left(1+k_{FF}\right)}^{2}}{1-k_{FF}}\left(h-2\tau \frac{1-k_{FF}}{1+k_{FF}}\right). \end{array} \end{array}$$

Finally, the string stability condition for CACC to satisfy (16), can be summarized as follows:

(24) $$\begin{array}{cc} \; & \left(c1\right)\;\left\{\begin{array}{c} 1-k_{FF}^{2}>0,\\ hk_{p}+\left(1-k_{FF}\right)k_{d}\leq \frac{1}{2m\tau }\left(1-k_{FF}^{2}\right),\\ hk_{p}+\left(1-k_{FF}\right)k_{d}\geq \frac{1}{mh}{\left(1-k_{FF}\right)}^{2}+\frac{1+k_{FF}}{2}hk_{p}, \end{array}\right.\\ \; & \mathrm{or}\\ \; & \left(c2\right)\;\left\{\begin{array}{c} 1-k_{FF}^{2}>0,\\ hk_{p}+\left(1-k_{FF}\right)k_{d}>\frac{1}{2m\tau }\left(1-k_{FF}^{2}\right),\\ {\left(k_{d}-\left(\frac{1+k_{FF}}{2m\tau }+\frac{k_{FF}}{1-k_{FF}}hk_{p}\right)\right)}^{2}\leq \frac{k_{p}}{m\tau }\frac{{\left(1+k_{FF}\right)}^{2}}{1-k_{FF}}\left(h-2\tau \frac{1-k_{FF}}{1+k_{FF}}\right). \end{array}\right. \end{array}$$

**Remark** **1.** *It is worth noting that by setting the feedforward gain $k_{FF}=0$, the proposed CACC controller reduces to a conventional ACC controller, as analyzed in [1]. In this case, the feedforward path is effectively removed, and the control input relies solely on the feedback PD controller. Consequently, the stability conditions in Table 1 reduce to those corresponding to the ACC case, and our derived conditions coincide with the analytical results previously established in [1]. This special case highlights the consistency of our framework with existing ACC results and demonstrates its generality in accommodating both ACC and CACC architectures under a unified analysis.*

**Remark** **2.** *If a CACC-based platoon system is both individually vehicle stable and string stable, then the time gap h and the time constant τ must satisfy the following condition:*

(25) $$\begin{array}{c} h\geq 2\tau \frac{1-k_{FF}}{1+k_{FF}}\;\left(\mathrm{or}\;h\left(1+k_{FF}\right)\geq 2\tau \left(1-k_{FF}\right)\right), \end{array}$$

*where the feedforward gain $k_{FF}$ is bounded by $-1<k_{FF}<1$, as required by condition *(19)*. In contrast to the ACC case, where $k_{FF}=0$ in *(25)* and the time gap must satisfy h≥2τ as discussed in [1], the CACC framework with $-1<k_{FF}<1$ permits the selection of a time gap h smaller than twice the plant time constant τ. In particular, when the feedforward gain is chosen sufficiently close to one (i.e., $k_{FF}\approx 1$), the required headway distance determined by the time gap h can be significantly reduced. In the extreme case where $k_{FF}\to 1$, the time gap h can approach zero. This flexibility confirms that a CACC-based system permits a much broader range for selecting h, compared to conventional ACC. From a design perspective, an engineer may first determine the time gap h according to system specifications, and then choose an appropriate $k_{FF}$ accordingly. This clearly illustrates that CACC is capable of overcoming the fundamental limitation of ACC in maintaining short inter-vehicle distances. The rationale behind this relaxed time gap condition is detailed below. First, under the string stability condition (c1) in *(24)*, it follows that*

(26a) $$\begin{array}{cc} \frac{1}{2m\tau }\left(1-k_{FF}^{2}\right) & \geq \frac{1}{mh}{\left(1-k_{FF}\right)}^{2}+\frac{1+k_{FF}}{2}hk_{p}, \end{array}$$

(26b) $$\begin{array}{cc} \frac{1}{2m\tau }\left(1-k_{FF}^{2}\right)-\frac{1}{mh}{\left(1-k_{FF}\right)}^{2} & \geq \frac{1+k_{FF}}{2}hk_{p}>0, \end{array}$$

(26c) $$\begin{array}{cc} \frac{1}{mh\tau }\left(1-k_{FF}\right)\left(h-2\tau \frac{1-k_{FF}}{1+k_{FF}}\right) & \geq hk_{p}>0, \end{array}$$

(26d) $$\begin{array}{cc} h-2\tau \frac{1-k_{FF}}{1+k_{FF}} & >0\;(\mathrm{or}\;h\left(1+k_{FF}\right)-2\tau \left(1-k_{FF}\right)>0). \end{array}$$

*Second, if the string stability condition (c2) in *(24)* holds, it follows that*

(27) $$\begin{array}{c} \begin{array}{cc} \frac{k_{p}}{m\tau }\frac{{\left(1+k_{FF}\right)}^{2}}{1-k_{FF}}\left(h-2\tau \frac{1-k_{FF}}{1+k_{FF}}\right) & \geq {\left(k_{d}-\left(\frac{1+k_{FF}}{2m\tau }+\frac{k_{FF}}{1-k_{FF}}hk_{p}\right)\right)}^{2}\geq 0,\\ h-2\tau \frac{1-k_{FF}}{1+k_{FF}} & \geq 0\;(\mathrm{or}\;h\left(1+k_{FF}\right)-2\tau \left(1-k_{FF}\right)\geq 0). \end{array} \end{array}$$

*It is worth noting that in both derivations, the individual vehicle stability condition *(15a)*, the basic assumptions h>0, τ>0, and the constraint $-1<k_{FF}<1$ are used as prerequisites. Since *(27)* allows equality in the condition $h-2\tau \frac{1-k_{FF}}{1+k_{FF}}\geq 0$, while *(26)* requires the strict inequality $h-2\tau \frac{1-k_{FF}}{1+k_{FF}}>0$ as a necessary condition, we consistently set $h-2\tau \frac{1-k_{FF}}{1+k_{FF}}>0$ in both cases to eliminate borderline ambiguity and preserve analytical consistency.*

### 3.4. String Stability for CACC with Communication Delays

In real-world implementations, communication delays are inevitable, which calls for a more rigorous string stability analysis that explicitly considers such delays. Specifically, we consider a constant communication delay of θ seconds, during which the desired acceleration command from the preceding vehicle becomes available to the ego vehicle. With this delay, the controller (8) with feedforward compensation is modified as follows:

(28) $$\begin{array}{c} \;u_{i}\left(t\right)=a_{d,i}\left(t\right)=k_{FF}a_{d,i-1}\left(t-\theta \right)+k_{p}\left(x_{i-1}\left(t\right)-x_{i}\left(t\right)-hv_{i}\left(t\right)\right)+k_{d}\left(v_{i-1}\left(t\right)-v_{i}\left(t\right)\right). \end{array}$$

Transforming Equation (28) into the Laplace domain yields the following:

$$\begin{array}{c} U_{i}\left(s\right)=k_{FF}e^{-\theta s}U_{i-1}\left(s\right)+k_{p}\left(X_{i-1}\left(s\right)-X_{i}\left(s\right)-hsX_{i}\left(s\right)\right)+k_{d}\left(sX_{i-1}\left(s\right)-sX_{i}\left(s\right)\right). \end{array}$$

Following the same derivation as in the previous subsection, the transfer function from $U_{i-1}\left(s\right)$ to $U_{i}\left(s\right)$, which remains independent of the vehicle index *i*, becomes

$$\begin{array}{c} \Gamma_{\theta }\left(s\right):=\frac{U_{i}\left(s\right)}{U_{i-1}\left(s\right)}=\frac{\tau k_{FF}e^{-\theta s}s^{3}+k_{FF}e^{-\theta s}s^{2}+mk_{d}s+mk_{p}}{\tau s^{3}+s^{2}+m\left(hk_{p}+k_{d}\right)s+mk_{p}}, \end{array}$$

which corresponds to the original transfer function in (10) with $k_{FF}$ replaced by $k_{FF}e^{-\theta s}$. Note that the exponential term $e^{-j\theta \omega }$ can be computed by

$$\begin{array}{c} e^{-j\theta \omega }=\cos\left(\theta \omega \right)-j\sin\left(\theta \omega \right). \end{array}$$

For string stability, the magnitude of Γ(jω) must satisfy the following:

$$\begin{array}{c} \begin{array}{cc} \; & {\left|\Gamma_{\theta }\left(j\omega \right)\right|}^{2}\\ \; & =\frac{{\left(mk_{p}-k_{FF}\omega^{2}\cos\left(\theta \omega \right)-\tau k_{FF}\omega^{3}\sin\left(\theta \omega \right)\right)}^{2}+{\left(mk_{d}\omega +k_{FF}\omega^{2}\sin\left(\theta \omega \right)-\tau k_{FF}\omega^{3}\cos\left(\theta \omega \right)\right)}^{2}}{{\left(mk_{p}-\omega^{2}\right)}^{2}+{\left(m\left(hk_{p}+k_{d}\right)\omega -\tau \omega^{3}\right)}^{2}}\\ \; & \leq 1,\;\forall \omega , \end{array} \end{array}$$

which is equivalently rewritten as follows:

$$\begin{array}{cc} \tau^{2}\left(1-k_{FF}^{2}\right)\omega^{6} & +\left(\left(1-k_{FF}^{2}\right)-2m\tau \left(hk_{p}+k_{d}\right)+2m\tau k_{FF}k_{d}\cos\left(\theta \omega \right)\right)\omega^{4}\\ \; & +2mk_{FF}\left(\tau k_{p}-k_{d}\right)\omega^{3}\sin\left(\theta \omega \right)\\ \; & +\left(m^{2}{\left(hk_{p}+k_{d}\right)}^{2}-2mk_{p}+2mk_{FF}k_{p}\cos\left(\theta \omega \right)-m^{2}k_{d}^{2}\right)\omega^{2}\geq 0,\;\forall \omega \geq 0. \end{array}$$

Assuming the delay is small (i.e., θ≪1), the trigonometric term can be approximated as follows:

$$\begin{array}{c} \cos\left(\theta \omega \right)\approx 1-\frac{\theta^{2}\omega^{2}}{2}\;\mathrm{and}\;\sin\left(\theta \omega \right)\approx \theta \omega , \end{array}$$

which leads to

$$\begin{array}{cc} \; & \left(\tau^{2}\left(1-k_{FF}^{2}\right)-m\tau k_{FF}k_{d}\theta^{2}\right)\omega^{6}\\ \; & +\left(\left(1-k_{FF}^{2}\right)-2m\tau \left(hk_{p}+\left(1-k_{FF}\right)k_{d}\right)-mk_{FF}k_{p}\theta^{2}+2mk_{FF}\left(\tau k_{p}-k_{d}\right)\theta \right)\omega^{4}\\ \; & +\left(m^{2}{\left(hk_{p}+k_{d}\right)}^{2}-2m\left(1-k_{FF}\right)k_{p}-m^{2}k_{d}^{2}\right)\omega^{2}\geq 0,\;\forall \omega \geq 0. \end{array}$$

With $\chi :=\omega^{2}$, the inequality can be rewritten into the following quadratic form:

$$\begin{array}{cc} f_{\theta }\left(\chi \right)= & a_{\theta }\chi^{2}+b_{\theta }\chi +c_{\theta }\\ = & \left(\tau^{2}\left(1-k_{FF}^{2}\right)-m\tau k_{FF}k_{d}\theta^{2}\right)\chi^{2}\\ \; & +\left(\left(1-k_{FF}^{2}\right)-2m\tau \left(hk_{p}+\left(1-k_{FF}\right)k_{d}\right)-mk_{FF}k_{p}\theta^{2}+2mk_{FF}\left(\tau k_{p}-k_{d}\right)\theta \right)\chi \\ \; & +\left(m^{2}{\left(hk_{p}+k_{d}\right)}^{2}-2m\left(1-k_{FF}\right)k_{p}-m^{2}k_{d}^{2}\right)\geq 0,\;\forall \chi \geq 0, \end{array}$$

where the coefficients and the discriminant are defined by

(29) $$\begin{array}{c} \begin{array}{cc} \; & a_{\theta }:=\tau^{2}\left(1-k_{FF}^{2}\right)-m\tau k_{FF}k_{d}\theta^{2},\\ \; & b_{\theta }:=\left(1-k_{FF}^{2}\right)-2m\tau \left(hk_{p}+\left(1-k_{FF}\right)k_{d}\right)-mk_{FF}k_{p}\theta^{2}+2mk_{FF}\left(\tau k_{p}-k_{d}\right)\theta ,\\ \; & c_{\theta }:=m^{2}{\left(hk_{p}+k_{d}\right)}^{2}-2m\left(1-k_{FF}\right)k_{p}-m^{2}k_{d}^{2},\\ \; & \Delta_{\theta }:=b_{\theta }^{2}-4a_{\theta }c_{\theta }. \end{array} \end{array}$$

The system is string stable if and only if one of the following conditions is satisfied:

(c_*θ*_1) $a_{\theta }>0$ and $b_{\theta }\geq 0$ and $c_{\theta }\geq 0$,
(c_*θ*_2) $a_{\theta }>0$ and $b_{\theta }<0$ and $\Delta_{\theta }\leq 0$.

Due to the complexity of the resulting expressions, it is difficult to extract intuitive or practical design guidelines directly from conditions ($c_{\theta }\;$1) and ($c_{\theta }\;$2) above. However, it is still possible to draw meaningful insights by comparing the present case with the communication delay to the delay-free scenario previously analyzed in Section 3.3.

**Remark** **3.** *Assume that there is no communication delay (θ=0) and that the control gains $k_{FF}^{\mathrm{old}}$, $k_{p}^{\mathrm{old}}$, and $k_{d}^{\mathrm{old}}$ have been selected according to the design guidelines to be presented in Section 4. Let the corresponding coefficients in *(17)* be denoted by $a^{\mathrm{old}},b^{\mathrm{old}},c^{\mathrm{old}}$, such that the resulting quadratic function satisfies*

$$\begin{array}{c} f^{\mathrm{old}}\left(\chi \right)=a^{\mathrm{old}}\chi^{2}+b^{\mathrm{old}}\chi +c^{\mathrm{old}}\geq 0\;\mathrm{for}\;\mathrm{all}\;\chi \geq 0. \end{array}$$

*Now consider the case with a relatively small communication delay θ>0. When the same control gains are applied, the corresponding coefficients *(29)* in the presence of delay, denoted as $a_{\theta }^{\mathrm{old}},b_{\theta }^{\mathrm{old}},c_{\theta }^{\mathrm{old}}$, can be expressed as follows:*

$$\begin{array}{cc} a_{\theta }^{\mathrm{old}} & =a^{\mathrm{old}}-m\tau k_{FF}^{\mathrm{old}}k_{d}^{\mathrm{old}}\theta^{2},\\ b_{\theta }^{\mathrm{old}} & =b^{\mathrm{old}}-mk_{FF}^{\mathrm{old}}k_{p}^{\mathrm{old}}\theta^{2}+2mk_{FF}^{\mathrm{old}}\left(\tau k_{p}^{\mathrm{old}}-k_{d}^{\mathrm{old}}\right)\theta ,\\ c_{\theta }^{\mathrm{old}} & =c^{\mathrm{old}}. \end{array}$$

*Among these, $b_{\theta }^{\mathrm{old}}$ is the most sensitive to delay, primarily due to the linear term $2mk_{FF}^{\mathrm{old}}\left(\tau k_{p}^{\mathrm{old}}-k_{d}^{\mathrm{old}}\right)\theta$, whereas $a_{\theta }^{\mathrm{old}}$ and $c_{\theta }^{\mathrm{old}}$ remain nearly unchanged for small θ. A simple yet sufficient condition for string stability in the presence of delay is that the coefficients satisfy*

$$\begin{array}{c} a_{\theta }^{\mathrm{old}}\approx a^{\mathrm{old}},\;b_{\theta }^{\mathrm{old}}\geq b^{\mathrm{old}},\;c_{\theta }^{\mathrm{old}}\approx c^{\mathrm{old}}, \end{array}$$

*which leads to*

$$\begin{array}{c} f_{\theta }^{\mathrm{old}}\left(\chi \right)=a_{\theta }^{\mathrm{old}}\chi^{2}+b_{\theta }^{\mathrm{old}}\chi +c_{\theta }^{\mathrm{old}}\geq f^{\mathrm{old}}\left(\chi \right)\geq 0\;\mathrm{for}\;\mathrm{all}\;\chi \geq 0. \end{array}$$

*Hence, it is desirable that $\tau k_{p}^{\mathrm{old}}-k_{d}^{\mathrm{old}}\geq 0$, or alternatively, $\tau k_{p}^{\mathrm{old}}-k_{d}^{\mathrm{old}}\approx 0$, so that $b_{\theta }^{\mathrm{old}}\geq b^{\mathrm{old}}$; that is, $2mk_{FF}^{\mathrm{old}}\left(\tau k_{p}^{\mathrm{old}}-k_{d}^{\mathrm{old}}\right)\theta \geq 0$.*

*With this consideration, we proceed to design the new control gains $k_{FF}^{\mathrm{new}}$, $k_{p}^{\mathrm{new}}$, and $k_{d}^{\mathrm{new}}$ by slightly adjusting the old ones. Let the resulting coefficients without delay be $a^{\mathrm{new}}$, $b^{\mathrm{new}}$, $c^{\mathrm{new}}$, and those with delay be $a_{\theta }^{\mathrm{new}}$, $b_{\theta }^{\mathrm{new}}$, $c_{\theta }^{\mathrm{new}}$. First, we check whether the coefficient $a_{\theta }^{\mathrm{old}}=a^{\mathrm{old}}-m\tau k_{FF}^{\mathrm{old}}k_{d}^{\mathrm{old}}\theta^{2}$ remains positive. If $a_{\theta }^{\mathrm{old}}>0$, we set $k_{FF}^{\mathrm{new}}=k_{FF}^{\mathrm{old}}$. Otherwise, we slightly decrease the feedforward gain as follows:*

$$k_{FF}^{\mathrm{new}}=k_{FF}^{\mathrm{old}}-\epsilon_{FF},\;\mathrm{for}\;\mathrm{some}\;\mathrm{small}\;\epsilon_{FF}>0.$$

*Note that reducing $k_{FF}$ may require increasing the headway time gap h, as indicated by the lower bound in (38). Second, to exploit the most delay-sensitive term $2mk_{FF}^{\mathrm{new}}\left(\tau k_{p}^{\mathrm{new}}-k_{d}^{\mathrm{new}}\right)\theta$ in $b_{\theta }^{\mathrm{new}}$, it is beneficial to choose $k_{d}^{\mathrm{new}}$ such that $\tau k_{p}^{\mathrm{new}}-k_{d}^{\mathrm{new}}\geq 0$, or alternatively $\tau k_{p}^{\mathrm{new}}-k_{d}^{\mathrm{new}}\approx 0$. Thus, we choose the derivative gain*

$$k_{d}^{\mathrm{new}}=k_{d}^{\mathrm{old}}-\epsilon_{d},\;\mathrm{for}\;\mathrm{some}\;\epsilon_{d}>0,$$

*while keeping the proportional gain $k_{p}^{\mathrm{new}}=k_{p}^{\mathrm{old}}$ unchanged. In other words, one may select $k_{d}^{\mathrm{new}}$ as small as possible around $\tau k_{p}^{\mathrm{new}}$, within the admissible range of $k_{d}$ values derived without considering the delay, as presented in Section 4.3. With the above choices, it is confirmed for the no-delay case that*

$$f^{\mathrm{new}}\left(\chi \right)=a^{\mathrm{new}}\chi^{2}+b^{\mathrm{new}}\chi +c^{\mathrm{new}}\geq 0\;\mathrm{for}\;\mathrm{all}\;\chi \geq 0.$$

*However, such a selection may potentially violate the string stability conditions ($c_{\theta }$1) and ($c_{\theta }$2) for the given delay θ; that is, the following inequality for the delayed case may not be satisfied:*

$$f_{\theta }^{\mathrm{new}}\left(\chi \right)=a_{\theta }^{\mathrm{new}}\chi^{2}+b_{\theta }^{\mathrm{new}}\chi +c_{\theta }^{\mathrm{new}}\geq 0\;\mathrm{for}\;\mathrm{all}\;\chi \geq 0.$$

*If this is the case, it becomes necessary to iteratively adjust $k_{p}^{\mathrm{new}}$ in either direction (i.e., increase or decrease from $k_{p}^{\mathrm{old}}$) and modify $k_{d}^{\mathrm{new}}$ accordingly, such that the condition $\tau k_{p}^{\mathrm{new}}-k_{d}^{\mathrm{new}}\geq 0$ holds, or alternatively $\tau k_{p}^{\mathrm{new}}-k_{d}^{\mathrm{new}}\approx 0$. This process requires iterative tuning to identify a suitable combination that satisfies the string stability condition in the presence of delay. Lastly, if no feasible combination of $k_{FF}^{\mathrm{new}}$, $k_{p}^{\mathrm{new}}$, and $k_{d}^{\mathrm{new}}$ satisfies the string stability condition in the presence of delay, the headway time gap h can be increased, and the above procedure repeated.*

### 3.5. String Stability for CACC with Feedforward Control Using the Actual Acceleration

When the desired acceleration is unavailable or not shared, particularly if the preceding vehicle is manually driven, the actual acceleration may nevertheless be obtained via V2V communication or on-board estimation in some practical CACC implementations. This subsection considers such a scenario, where the actual acceleration is used in the feedforward term, and follows a procedure similar to the previous one, except that the feedforward term uses the *actual* acceleration of the preceding vehicle instead of the *desired* acceleration. Specifically, the control input in (8) is modified by replacing $a_{d,i-1}\left(t\right)$ with $a_{i-1}\left(t\right)$, resulting in the following control law:

(30) $$\begin{array}{c} u_{i}\left(t\right)=a_{d,i}\left(t\right)=k_{FF}a_{i-1}\left(t\right)+k_{p}\left(x_{i-1}\left(t\right)-x_{i}\left(t\right)-hv_{i}\left(t\right)\right)+k_{d}\left(v_{i-1}\left(t\right)-v_{i}\left(t\right)\right). \end{array}$$

In the Laplace domain, Equation (30) can be expressed as follows:

$$\begin{array}{c} \begin{array}{cc} U_{i}\left(s\right) & =k_{FF}A_{i-1}\left(s\right)+k_{p}\left(X_{i-1}\left(s\right)-X_{i}\left(s\right)-hsX_{i}\left(s\right)\right)+k_{d}\left(sX_{i-1}\left(s\right)-sX_{i}\left(s\right)\right)\\ \; & =\frac{mk_{FF}}{\tau s+1}U_{i-1}\left(s\right)+k_{p}P\left(s\right)\left(U_{i-1}\left(s\right)-U_{i}\left(s\right)-hsU_{i}\left(s\right)\right)+k_{d}sP\left(s\right)\left(U_{i-1}\left(s\right)-U_{i}\left(s\right)\right). \end{array} \end{array}$$

Following the same approach as in the previous subsection, the transfer function from $U_{i-1}\left(s\right)$ to $U_{i}\left(s\right)$, which is independent of *i*, is given by the following:

$$\begin{array}{c} \begin{array}{cc} \Gamma_{a}\left(s\right):=\frac{U_{i}\left(s\right)}{U_{i-1}\left(s\right)} & =\frac{\frac{mk_{FF}}{\tau s+1}+P\left(s\right)\left(k_{d}s+k_{p}\right)}{1+P\left(s\right)\left(hk_{p}s+k_{d}s+k_{p}\right)}=\frac{mk_{FF}s^{2}+mk_{d}s+mk_{p}}{\tau s^{3}+s^{2}+m\left(hk_{p}+k_{d}\right)s+mk_{p}}. \end{array} \end{array}$$

For string stability, the magnitude of Γ(jω) must satisfy the following:

$$\begin{array}{c} {\left|\Gamma_{a}\left(j\omega \right)\right|}^{2}=\frac{{\left(mk_{p}-mk_{FF}\omega^{2}\right)}^{2}+{\left(mk_{d}\omega \right)}^{2}}{{\left(mk_{p}-\omega^{2}\right)}^{2}+{\left(m\left(hk_{p}+k_{d}\right)\omega -\tau \omega^{3}\right)}^{2}}\leq 1,\;\forall \omega \geq 0, \end{array}$$

which is equivalently rewritten as follows:

$$\begin{array}{cc} \tau^{2}\omega^{6} & +\left(\left(1-m^{2}k_{FF}^{2}\right)-2m\tau \left(hk_{p}+k_{d}\right)\right)\omega^{4}\\ \; & +\left(m^{2}{\left(hk_{p}+k_{d}\right)}^{2}-2m\left(1-mk_{FF}\right)k_{p}-m^{2}k_{d}^{2}\right)\omega^{2}\geq 0,\;\forall \omega \geq 0. \end{array}$$

Introducing the substitution $\chi :=\omega^{2}$, the inequality becomes a quadratic form:

$$\begin{array}{cc} \tau^{2}\chi^{2} & +\left(\left(1-m^{2}k_{FF}^{2}\right)-2m\tau \left(hk_{p}+k_{d}\right)\right)\chi \\ \; & +\left(m^{2}{\left(hk_{p}+k_{d}\right)}^{2}-2m\left(1-mk_{FF}\right)k_{p}-m^{2}k_{d}^{2}\right)\geq 0,\;\forall \chi \geq 0. \end{array}$$

Let us define the coefficients:

$$\begin{array}{cc} \; & a_{a}:=\tau^{2},\\ \; & b_{a}:=\left(1-m^{2}k_{FF}^{2}\right)-2m\tau \left(hk_{p}+k_{d}\right),\\ \; & c_{a}:=m^{2}{\left(hk_{p}+k_{d}\right)}^{2}-2m\left(1-mk_{FF}\right)k_{p}-m^{2}k_{d}^{2}, \end{array}$$

and the discriminant:

$$\Delta_{a}:=b_{a}^{2}-4a_{a}c_{a}.$$

The system is string stable if and only if one of the following conditions holds:

(c_a_1) $b_{a}\geq 0$ and $c_{a}\geq 0$,
(c_a_2) $b_{a}<0$ and $\Delta_{a}\leq 0$.

Condition ($c_{a}\;$1) leads to the following inequalities:

$$\begin{array}{cc} hk_{p}+k_{d}\leq \frac{1}{2m\tau }\left(1-m^{2}k_{FF}^{2}\right)\;\mathrm{and}\; & m^{2}\left(h^{2}k_{p}^{2}+2hk_{p}k_{d}\right)-2m\left(1-mk_{FF}\right)k_{p}\geq 0,\\ hk_{p}+k_{d}\leq \frac{1}{2m\tau }\left(1-m^{2}k_{FF}^{2}\right)\;\mathrm{and}\; & hk_{p}+2k_{d}\geq \frac{2}{mh}\left(1-mk_{FF}\right),\\ \frac{1}{mh}\left(1-mk_{FF}\right)+\frac{1}{2}hk_{p}\leq & hk_{p}+k_{d}\leq \frac{1}{2m\tau }\left(1-m^{2}k_{FF}^{2}\right). \end{array}$$

Condition ($c_{a}\;$2) is given by the following:

$$\begin{array}{c} \begin{array}{cc} \; & hk_{p}+k_{d}>\frac{1}{2m\tau }\left(1-m^{2}k_{FF}^{2}\right)\;\mathrm{and}\\ \; & \;\;\;{\left(\left(1-m^{2}k_{FF}^{2}\right)-2m\tau k_{d}\right)}^{2}+4m^{2}h^{2}\tau^{2}k_{p}^{2}-4mh\tau k_{p}\left(\left(1-m^{2}k_{FF}^{2}\right)-2m\tau k_{d}\right)\\ \; & \;\;\;-4\tau^{2}\left(m^{2}h^{2}k_{p}^{2}+2m^{2}hk_{p}k_{d}-2m\left(1-mk_{FF}\right)k_{p}\right)\leq 0,\\ \; & hk_{p}+k_{d}>\frac{1}{2m\tau }\left(1-m^{2}k_{FF}^{2}\right)\;\mathrm{and}\\ \; & \;\;\;{\left(\left(1-m^{2}k_{FF}^{2}\right)-2m\tau k_{d}\right)}^{2}\leq 4m\tau k_{p}\left(h\left(1-m^{2}k_{FF}^{2}\right)-2\tau \left(1-mk_{FF}\right)\right),\\ \; & hk_{p}+k_{d}>\frac{1}{2m\tau }\left(1-m^{2}k_{FF}^{2}\right)\;\mathrm{and}\\ \; & \;\;\;{\left(k_{d}-\frac{1}{2m\tau }\left(1-m^{2}k_{FF}^{2}\right)\right)}^{2}\leq \frac{k_{p}}{m\tau }\left(1-m^{2}k_{FF}^{2}\right)\left(h-\frac{2\tau }{1+mk_{FF}}\right). \end{array} \end{array}$$

Finally, the string stability condition is satisfied if and only if either $\left(c_{a}\;1\right)$ or $(c_{a}\;$2) holds:

(31) $$\begin{array}{cc} \; & (c_{a}\;1)\;\left\{\begin{array}{c} hk_{p}+k_{d}\leq \frac{1}{2m\tau }\left(1-m^{2}k_{FF}^{2}\right),\\ hk_{p}+k_{d}\geq \frac{1}{mh}\left(1-mk_{FF}\right)+\frac{1}{2}hk_{p}, \end{array}\right.\\ \; & \mathrm{or}\\ \; & (c_{a}\;2)\;\left\{\begin{array}{c} hk_{p}+k_{d}>\frac{1}{2m\tau }\left(1-m^{2}k_{FF}^{2}\right),\\ {\left(k_{d}-\frac{1}{2m\tau }\left(1-m^{2}k_{FF}^{2}\right)\right)}^{2}\leq \frac{k_{p}}{m\tau }\left(1-m^{2}k_{FF}^{2}\right)\left(h-\frac{2\tau }{1+mk_{FF}}\right). \end{array}\right. \end{array}$$

**Remark** **4.** *Even though the condition $1-m^{2}k_{FF}^{2}>0$, which is equivalent to *(19)* with m=1, does not explicitly appear in the string stability condition *(31)*, it must still be satisfied in this case. Note that individual vehicle stability requires the condition given in *(15)*, which implies*

$$hk_{p}+k_{d}>hk_{p}+\left(\tau -h\right)k_{p}=\tau k_{p}>0.$$

*This indicates that the first inequality of condition ($c_{a}\;$1) in *(31)* cannot hold if $1-m^{2}k_{FF}^{2}\leq 0$. Now, for the sake of contradiction, suppose that both the string stability condition ($c_{a}\;$2) in *(31)* and the individual vehicle stability condition in *(15)* are satisfied, while $1-m^{2}k_{FF}^{2}\leq 0$. Then, it easily follows that*

(32) $$\begin{array}{c} h-\frac{2\tau }{1+mk_{FF}}\leq 0. \end{array}$$

*First, consider the case where $k_{FF}\leq -1/m$. In this case, inequality *(32)* implies $h\leq \frac{2\tau }{1+mk_{FF}}\leq 0$, which contradicts the fact that h>0. Next, consider the case where $k_{FF}\geq 1/m$. From inequality *(32)*, it follows that $h\leq \frac{2\tau }{1+mk_{FF}}\leq \tau$, which implies that τ−h≥0. Combined with the individual vehicle stability condition in *(15)*, we obtain $k_{d}>\left(\tau -h\right)k_{p}\geq 0$. Substituting this into the second inequality of condition ($c_{a}\;$2) in *(31)*, we obtain the following:*

$$\begin{array}{cc} {\left(\left(\tau -h\right)k_{p}+\frac{1}{2m\tau }\left(m^{2}k_{FF}^{2}-1\right)\right)}^{2}\; & <\frac{k_{p}}{m\tau }\left(m^{2}k_{FF}^{2}-1\right)\left(\tau -h+\frac{1-mk_{FF}}{1+mk_{FF}}\tau \right),\\ {\left(\tau -h\right)}^{2}k_{p}^{2}+\frac{1}{4m^{2}\tau^{2}}{\left(m^{2}k_{FF}^{2}-1\right)}^{2}\; & <-\frac{k_{p}}{m}{\left(mk_{FF}-1\right)}^{2}, \end{array}$$

*where the left-hand side is clearly non-negative and the right-hand side is non-positive. This contradiction shows that the assumption $1-m^{2}k_{FF}^{2}\leq 0$ cannot hold. Therefore, we conclude that*

(33) $$\begin{array}{c} 1-m^{2}k_{FF}^{2}>0\;\left(\mathrm{or}\;-1/m<k_{FF}<1/m\right). \end{array}$$

**Remark** **5.** *If a CACC-based platoon system uses the actual acceleration of the preceding vehicle for the feedforward control, then the time gap h and the plant time constant τ must satisfy the following necessary condition:*

(34) $$\begin{array}{c} h\geq \frac{2\tau }{1+mk_{FF}}\;\left(\mathrm{or}\;h\left(1+mk_{FF}\right)\geq 2\tau \right), \end{array}$$

*where the feedforward gain $k_{FF}$ must lie within the interval $-1/m<k_{FF}<1/m$, as required by condition *(33)* in Remark 4. Notably, as $mk_{FF}$ approaches one (i.e., $k_{FF}\to 1/m$), the minimum admissible time gap h decreases and converges to the time constant τ of the vehicle dynamics. Compared to the ACC case with h≥2τ analyzed in [1], the result in *(34)* permits a smaller minimum time gap. However, it is still more conservative than the case where the desired acceleration is used in the feedforward path, as discussed in *(25)* in Remark 2. The derivation underlying the time gap condition in *(34)* is outlined as follows. First, under the string stability condition ($c_{a}\;$1) in *(31)*, we obtain the following sequence of inequalities:*

(35a) $$\begin{array}{cc} \frac{1}{2m\tau }\left(1-m^{2}k_{FF}^{2}\right) & \geq \frac{1}{mh}\left(1-mk_{FF}\right)+\frac{1}{2}hk_{p}, \end{array}$$

(35b) $$\begin{array}{cc} \frac{1}{2m\tau }\left(1-m^{2}k_{FF}^{2}\right)-\frac{1}{mh}\left(1-mk_{FF}\right) & \geq \frac{1}{2}hk_{p}>0, \end{array}$$

(35c) $$\begin{array}{cc} \frac{1}{mh\tau }\left(1-m^{2}k_{FF}^{2}\right)\left(h-\frac{2\tau }{1+mk_{FF}}\right) & \geq hk_{p}>0, \end{array}$$

(35d) $$\begin{array}{cc} h-\frac{2\tau }{1+mk_{FF}} & >0\;(\mathrm{or}\;h\left(1+mk_{FF}\right)-2\tau >0). \end{array}$$

*Next, when the string stability condition ($c_{a}\;$2) in *(31)* is satisfied, a similar requirement on the time gap arises:*

(36) $$\begin{array}{c} \begin{array}{cc} \frac{k_{p}}{m\tau }\left(1-m^{2}k_{FF}^{2}\right)\left(h-\frac{2\tau }{1+mk_{FF}}\right) & \geq {\left(k_{d}-\frac{1}{2m\tau }\left(1-m^{2}k_{FF}^{2}\right)\right)}^{2}\geq 0,\\ h-\frac{2\tau }{1+mk_{FF}} & \geq 0\;(\mathrm{or}\;h\left(1+mk_{FF}\right)-2\tau \geq 0). \end{array} \end{array}$$

*It is important to note that both derivations assume the individual vehicle stability condition in *(15a)*, along with the basic properties h>0, τ>0, and the gain constraint $-1/m<k_{FF}<1/m$. Since *(36)* permits equality in the time gap lower bound, while *(35)* requires a strict inequality, we consistently adopt the strict form $h-\frac{2\tau }{1+mk_{FF}}>0$ for both conditions to avoid edge cases and ensure analytical uniformity.*

## 4. Design of PD Control with Feedforward Compensation

Recall the control design objective stated in Problem 1 and the PD and feedforward control law introduced in (8):

$$\begin{array}{c} u_{i}\left(t\right)=k_{FF}u_{i-1}\left(t\right)+k_{p}\left(x_{i-1}\left(t\right)-x_{i}\left(t\right)-hv_{i}\left(t\right)\right)+k_{d}\left(v_{i-1}\left(t\right)-v_{i}\left(t\right)\right). \end{array}$$

**Figure 3:**
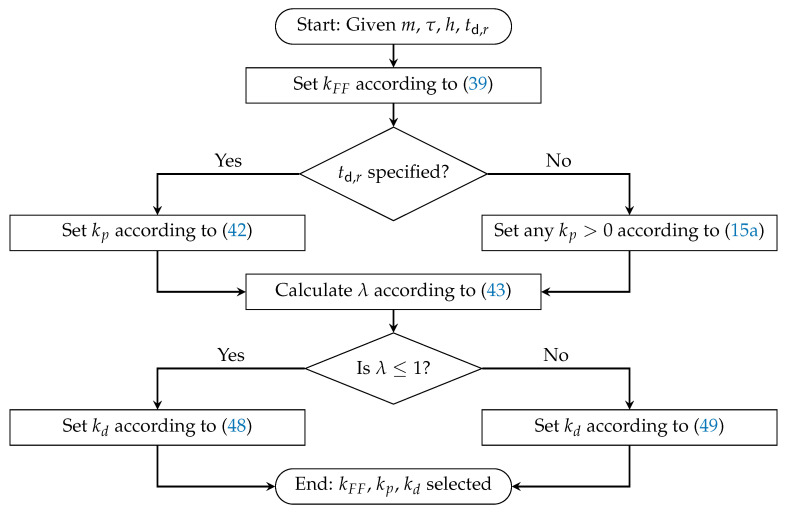
Flowchart illustrating the design guideline in Algorithm 1.

Based on the stability conditions developed in Section 3, we present a systematic procedure for selecting the feedforward gain $k_{FF}$ and the proportional–derivative gains $k_{p}$ and $k_{d}$ such that the closed-loop system satisfies both the individual vehicle stability condition (15) and the string stability condition (24). The complete controller design guideline is first summarized in Algorithm 1, and its procedural steps are illustrated in the flowchart shown in Figure 3. The correctness of Algorithm 1 is formally established in Theorem 1.
**Algorithm 1** Design guidelines for string stability and individual vehicle stability**Input:** *m*, τ, *h*, $t_{d,r}$**Output:** $k_{FF}$, $k_{p}$, $k_{d}$   1: Set $k_{FF}$ s.t. $\max\left\{-1+\frac{4\tau }{h+2\tau },\;0\right\}\leq k_{FF}<1$ (according to (39))   2: Set any positive value for $k_{p}$ s.t. $k_{p}>0$ (according to (15a))   3: **if ** the desired rise time is specified as $t_{d,r}$ **then**   4:      Set $k_{p}$ s.t. $k_{p}>\frac{1.8^{2}}{m\;t_{d,r}^{2}}$ (according to (42))   5: **end if**   6: Let $k_{p}=\frac{\lambda }{mh^{2}\tau }\left(1-k_{FF}\right)\left(h-2\tau \frac{1-k_{FF}}{1+k_{FF}}\right)$ (according to (43))   7: **if **
0<λ≤1
** then**   8:      Set $k_{d}\;s.t.\;k_{d}>\max\left\{\frac{1-k_{FF}}{mh}-\frac{\lambda (1-k_{FF})}{2mh\tau }\left(h-2\tau \frac{1-k_{FF}}{1+k_{FF}}\right),\;\left(\tau -h\right)k_{p}\right\}\;\&$   9:                       $k_{d}\leq \frac{1+k_{FF}}{2m\tau }+\frac{\lambda k_{FF}+\sqrt{\lambda }\left(1+k_{FF}\right)}{mh\tau }\left(h-2\tau \frac{1-k_{FF}}{1+k_{FF}}\right)$ (according to (48))  10: **else if **
λ>1
** then**  11:      Set $k_{d}$ s.t. $k_{d}>\max\left\{\frac{1+k_{FF}}{2m\tau }+\frac{\lambda k_{FF}-\sqrt{\lambda }\left(1+k_{FF}\right)}{mh\tau }\left(h-2\tau \frac{1-k_{FF}}{1+k_{FF}}\right),\;\left(\tau -h\right)k_{p}\right\}\;\&$  12:                       $k_{d}\leq \frac{1+k_{FF}}{2m\tau }+\frac{\lambda k_{FF}+\sqrt{\lambda }\left(1+k_{FF}\right)}{mh\tau }\left(h-2\tau \frac{1-k_{FF}}{1+k_{FF}}\right)$ (according to (49))  13: **end if**

**Theorem** **1.** *Consider a platoon system where each vehicle has identical longitudinal dynamics as described by *(3)*, and is equipped with a CACC controller of the form given in *(8)*. If the control gains $k_{p}$, $k_{d}$, and $k_{FF}$ are selected according to the design guideline outlined in Algorithm 1, then the platoon system under the CTG policy *(1)* is both string stable and individually vehicle stable.*

*Conversely, if any of the following conditions is satisfied:*
*(i) $k_{FF}\notin \left[-1+\frac{4\tau }{h+2\tau },\;1\right)$;*
*(ii) $k_{p}\leq 0$; or*
*(iii) $k_{d}$ is chosen outside the range specified in Algorithm 1, i.e., *(48)* for 0<λ≤1 and *(49)* for λ>1, then the PD and feedforward-based CACC platoon system does not achieve string stability or individual vehicle stability.*

**Proof.**  The proof follows from Section 4.1, Section 4.2 and Section 4.3. □

The subsequent subsections provide the theoretical background and detailed rationale that support the validity of both Algorithm 1 and Theorem 1, along with the systematic determination of controller gains. First, the feedforward gain $k_{FF}$ is selected based on the relationship between the time gap *h* and the plant time constant τ. Then, the proportional gain $k_{p}$ is chosen primarily to satisfy dynamic performance requirements, such as a specified rise time. Lastly, the derivative gain $k_{d}$ is tuned to ensure that both individual vehicle stability and string stability conditions are satisfied.

### 4.1. Determination of Feedforward Gain $k_{FF}$

We begin by determining the feedforward gain $k_{FF}$ from the vehicle time constant τ and the time gap *h*. This design step is guided by the necessary condition (25) presented in Remark 2, which is rewritten in the following inequality:

(37) $$\begin{array}{c} h\left(1+k_{FF}\right)-2\tau \left(1-k_{FF}\right)\geq 0. \end{array}$$

In this context, the time constant τ is a fixed parameter determined by the vehicle’s inherent longitudinal dynamics, whereas the time gap *h* is a tunable design variable specified by the CACC system designer. From inequality (37), a lower bound on the feedforward gain $k_{FF}$ is obtained as

(38) $$\begin{array}{c} -1+\frac{4\tau }{h+2\tau }=\frac{-h+2\tau }{h+2\tau }\leq k_{FF}. \end{array}$$

Moreover, to satisfy the string stability condition established in (19), $k_{FF}$ must also lie within the range

$$-1<k_{FF}<1.$$

Therefore, the final selection of the feedforward gain should satisfy the following inequality:

$$\begin{array}{c} -1+\frac{4\tau }{h+2\tau }\leq k_{FF}<1. \end{array}$$

As noted in Remark 2, the primary motivation for employing CACC instead of ACC is its capacity to overcome the inherent limitation of ACC, which requires a relatively long time gap h>2τ. Accordingly, CACC is typically applied in scenarios where the time gap satisfies h≤2τ. Notably, at the boundary case h=2τ, the feasible range for $k_{FF}$ derived from (38) simplifies to $0\leq k_{FF}$. Based on these observations, the final design guideline for selecting the feedforward gain $k_{FF}$ is proposed as

(39) $$\begin{array}{c} \max\left\{-1+\frac{4\tau }{h+2\tau },\;0\right\}\leq k_{FF}<1. \end{array}$$

### 4.2. Determination of Proportional Gain $k_{p}$

Recall the individual vehicle stability condition (15a), which requires $k_{p}>0$. Among the stability conditions, the inequality $k_{p}>0$ serves as the only independent requirement specifically imposed on the proportional gain $k_{p}$. Building on the basic requirement $k_{p}>0$, we provide a practical guideline for selecting the proportional gain to aid engineers in the design of CACC systems. This guideline is grounded in the dynamic characteristics of a standard second-order system:

(40) $$\begin{array}{c} L\left(s\right)=\frac{\omega_{n}^{2}}{s^{2}+2\zeta \omega_{n}s+\omega_{n}^{2}}, \end{array}$$

where $\omega_{n}$ and ζ denote the natural frequency and damping ratio, respectively. For such a system, the rise time $t_{r}$, defined as the time required for the output to rise from 10% to 90% of its steady-state value, can be approximated by

$$\begin{array}{c} t_{r}\approx \frac{1.8}{\omega_{n}}, \end{array}$$

as noted in [40].

Assuming ideal vehicle dynamics, i.e., τ=0, the longitudinal model simplifies to the following:

$$\begin{array}{c} P_{\mathrm{ideal}}\left(s\right)=\frac{m}{s^{2}}, \end{array}$$

which implies that the actual acceleration instantly matches the commanded acceleration without delay. Under this assumption, the transfer function G(s) in (11) simplifies to the following:

(41) $$\begin{array}{c} G_{\mathrm{ideal}}\left(s\right)=\frac{-hk_{FF}s+\left(\left(1-k_{FF}\right)-mhk_{d}\right)}{s^{2}+m\left(hk_{p}+k_{d}\right)s+mk_{p}}. \end{array}$$

Comparing the denominator of (41) with that of the standard second-order form (40), we identify the natural frequency as $\omega_{n}^{2}=mk_{p}$. Hence, to approximately achieve a desired rise time $t_{d,r}$, the proportional gain $k_{p}$ can be selected according to the following inequality:

(42) $$\begin{array}{c} \begin{array}{cc} t_{d,r} & \geq t_{r}\approx \frac{1.8}{\sqrt{mk_{p}}},\\ k_{p} & >\frac{1.8^{2}}{m\;t_{d,r}^{2}}. \end{array} \end{array}$$

It is important to note that the guideline above is derived under the idealized assumption of zero time constant (τ=0). Therefore, in practical implementation, an appropriate design margin should be incorporated to compensate for actuator delays and model uncertainties.

### 4.3. Determination of Derivative Gain $k_{d}$

Given that the proportional gain $k_{p}$ is designed to satisfy (42), it can be written as

(43) $$\begin{array}{c} k_{p}=\frac{\lambda }{mh^{2}\tau }\left(1-k_{FF}\right)\left(h-2\tau \frac{1-k_{FF}}{1+k_{FF}}\right), \end{array}$$

where the parameter λ>0 is implicitly determined by the other system parameters, along with the necessary stability condition $h-2\tau \frac{1-k_{FF}}{1+k_{FF}}>0$ stated in Remark 2.

First, consider the case where 0<λ≤1, and denote λ by α in this range. With

$$k_{p}=\frac{\alpha }{mh^{2}\tau }\left(1-k_{FF}\right)\left(h-2\tau \frac{1-k_{FF}}{1+k_{FF}}\right),\;\mathrm{where}\;0<\alpha \leq 1,$$

inequality (26c), which corresponds to condition (c1) in (24), is satisfied. Accordingly, the derivative gain $k_{d}$ should be selected to ensure that condition (c1) in (24) is satisfied. From inequality (21), the corresponding admissible range for $k_{d}$ can be derived as follows:

(44) $$\begin{array}{cc} \frac{1}{mh}{\left(1-k_{FF}\right)}^{2}+\frac{1+k_{FF}}{2}hk_{p} & \leq hk_{p}+\left(1-k_{FF}\right)k_{d}\leq \frac{1}{2m\tau }\left(1-k_{FF}^{2}\right),\\ \frac{1}{mh}{\left(1-k_{FF}\right)}^{2}-\frac{1-k_{FF}}{2}hk_{p} & \leq \left(1-k_{FF}\right)k_{d}\leq \frac{1}{2m\tau }\left(1-k_{FF}^{2}\right)-hk_{p},\\ \frac{1}{mh}\left(1-k_{FF}\right)-\frac{1}{2}hk_{p} & \leq k_{d}\leq \frac{1}{2m\tau }\left(1+k_{FF}\right)-\frac{1}{1-k_{FF}}hk_{p},\\ \frac{1-k_{FF}}{mh}-\frac{\alpha (1-k_{FF})}{2mh\tau }\left(h-2\tau \frac{1-k_{FF}}{1+k_{FF}}\right) & \leq k_{d}\leq \frac{1+k_{FF}}{2m\tau }-\frac{\alpha }{mh\tau }\left(h-2\tau \frac{1-k_{FF}}{1+k_{FF}}\right). \end{array}$$

Here, since α≤1, it holds that

$$\begin{array}{cc} \; & \left(\frac{1+k_{FF}}{2m\tau }-\frac{\alpha }{mh\tau }\left(h-2\tau \frac{1-k_{FF}}{1+k_{FF}}\right)\;\right)-\left(\frac{1-k_{FF}}{mh}-\frac{\alpha (1-k_{FF})}{2mh\tau }\left(h-2\tau \frac{1-k_{FF}}{1+k_{FF}}\right)\;\right)\\ \; & =\left(\frac{h(1+k_{FF})}{2mh\tau }-\frac{\alpha }{mh\tau }\left(h-2\tau \frac{1-k_{FF}}{1+k_{FF}}\right)\;\right)-\left(\frac{\tau (1-k_{FF})}{mh\tau }-\frac{\alpha (1-k_{FF})}{2mh\tau }\left(h-2\tau \frac{1-k_{FF}}{1+k_{FF}}\right)\;\right)\\ \; & =\frac{1+k_{FF}}{2mh\tau }\left(1-\alpha \right)\left(h-2\tau \frac{1-k_{FF}}{1+k_{FF}}\right)\geq 0, \end{array}$$

which implies that the inequality in (44) is feasible. Consequently, a derivative gain $k_{d}$ that satisfies (44) exists and can be appropriately selected. Now, let us derive the condition on $k_{d}$ required to satisfy condition (c2) in (24). From inequality (23), the bounds on $k_{d}$ are obtained as follows:

$$\begin{array}{c} \begin{array}{cc} \; & hk_{p}+\left(1-k_{FF}\right)k_{d}>\frac{1}{2m\tau }\left(1-k_{FF}^{2}\right)\;\mathrm{and}\\ \; & \;\;{\left(k_{d}-\left(\frac{1+k_{FF}}{2m\tau }+\frac{k_{FF}}{1-k_{FF}}hk_{p}\right)\right)}^{2}\leq \frac{k_{p}}{m\tau }\frac{{\left(1+k_{FF}\right)}^{2}}{1-k_{FF}}\left(h-2\tau \frac{1-k_{FF}}{1+k_{FF}}\right),\\ \; & k_{d}>\frac{1+k_{FF}}{2m\tau }-\frac{1}{1-k_{FF}}hk_{p}\;\mathrm{and}\\ \; & \;\;\frac{1+k_{FF}}{2m\tau }+\frac{k_{FF}}{1-k_{FF}}hk_{p}-\sqrt{\frac{k_{p}}{m\tau }\frac{{\left(1+k_{FF}\right)}^{2}}{1-k_{FF}}\left(h-2\tau \frac{1-k_{FF}}{1+k_{FF}}\right)}\leq k_{d}\\ \; & \;\;\;\;\;\;\leq \frac{1+k_{FF}}{2m\tau }+\frac{k_{FF}}{1-k_{FF}}hk_{p}+\sqrt{\frac{k_{p}}{m\tau }\frac{{\left(1+k_{FF}\right)}^{2}}{1-k_{FF}}\left(h-2\tau \frac{1-k_{FF}}{1+k_{FF}}\right)},\\ \; & k_{d}>\frac{1+k_{FF}}{2m\tau }-\frac{\alpha }{mh\tau }\left(h-2\tau \frac{1-k_{FF}}{1+k_{FF}}\right)\;\mathrm{and}\\ \; & \;\;\frac{1+k_{FF}}{2m\tau }+\frac{\alpha k_{FF}-\sqrt{\alpha }\left(1+k_{FF}\right)}{mh\tau }\left(h-2\tau \frac{1-k_{FF}}{1+k_{FF}}\right)\leq k_{d}\\ \; & \;\;\;\;\;\;\leq \frac{1+k_{FF}}{2m\tau }+\frac{\alpha k_{FF}+\sqrt{\alpha }\left(1+k_{FF}\right)}{mh\tau }\left(h-2\tau \frac{1-k_{FF}}{1+k_{FF}}\right), \end{array} \end{array}$$

(45) $$\begin{array}{c} \begin{array}{cc} \; & \frac{1\;+\;k_{FF}}{2m\tau }\;-\;\frac{\alpha }{mh\tau }\left(\;h\;-\;2\tau \frac{1-k_{FF}}{1+k_{FF}}\;\right)\;<k_{d}\leq \frac{1\;+\;k_{FF}}{2m\tau }+\frac{\alpha k_{FF}\;+\;\sqrt{\alpha }\left(1\;+\;k_{FF}\right)}{mh\tau }\left(\;h\;-\;2\tau \frac{1-k_{FF}}{1+k_{FF}}\;\right), \end{array} \end{array}$$

where the inequality $\alpha \leq \sqrt{\alpha }$, valid for 0<α≤1, is applied in the final step to ensure that $-\alpha \geq \alpha k_{FF}-\sqrt{\alpha }\left(1+k_{FF}\right)$. It is straightforward to verify that the upper bound exceeds the lower bound, confirming that an appropriate derivative gain $k_{d}$ can be selected to satisfy (45). Since the stability condition requires that either condition (c1) or (c2) holds, the admissible range of $k_{d}$ is characterized by the union of the bounds provided in (44) and (45) as follows:

(46) $$\begin{array}{c} \begin{array}{cc} \frac{1-k_{FF}}{mh} & -\frac{\alpha (1-k_{FF})}{2mh\tau }\left(h-2\tau \frac{1-k_{FF}}{1+k_{FF}}\right)\leq k_{d}\\ \; & \leq \frac{1+k_{FF}}{2m\tau }+\frac{\alpha k_{FF}+\sqrt{\alpha }\left(1+k_{FF}\right)}{mh\tau }\left(h-2\tau \frac{1-k_{FF}}{1+k_{FF}}\right). \end{array} \end{array}$$

Furthermore, it can be shown that a suitable $k_{d}$ can always be chosen to meet the condition (15b), as demonstrated below:

(47) $$\begin{array}{c} \begin{array}{cc} \; & \frac{1+k_{FF}}{2}h>0,\\ \; & \frac{1+k_{FF}}{2}h>\left(h-2\tau \frac{1-k_{FF}}{1+k_{FF}}\right)\left(-\alpha \frac{1-k_{FF}}{2}-\sqrt{\alpha }\left(1+k_{FF}\right)\right),\\ \; & \frac{1+k_{FF}}{2}h>\left(h-2\tau \frac{1-k_{FF}}{1+k_{FF}}\right)\left(\alpha \frac{1+k_{FF}}{2}-\alpha -\sqrt{\alpha }\left(1+k_{FF}\right)\right),\\ \; & \frac{1+k_{FF}}{2}h>\left(h-2\tau \frac{1-k_{FF}}{1+k_{FF}}\right)\left(\alpha \frac{\tau }{h}\left(1-k_{FF}\right)-\alpha -\sqrt{\alpha }\left(1+k_{FF}\right)\right),\\ \; & \frac{1+k_{FF}}{2}h>\left(h-2\tau \frac{1-k_{FF}}{1+k_{FF}}\right)\left(\alpha \left(\frac{\tau }{h}-1\right)\left(1-k_{FF}\right)-\alpha k_{FF}-\sqrt{\alpha }\left(1+k_{FF}\right)\right),\\ \; & \frac{1\;+\;k_{FF}}{2}h\;+\;\left(\alpha k_{FF}\;+\;\sqrt{\alpha }\left(1\;+\;k_{FF}\right)\right)\left(\;h\;-\;2\tau \frac{1\;-\;k_{FF}}{1\;+\;k_{FF}}\;\right)\;\;>\;\;\frac{\alpha }{h}\left(\tau \;-\;h\right)\left(1\;-\;k_{FF}\right)\left(\;h\;-\;2\tau \frac{1\;-\;k_{FF}}{1\;+\;k_{FF}}\;\right),\\ \; & \frac{1\;+\;k_{FF}}{2m\tau }\;+\;\frac{\alpha k_{FF}\;+\;\sqrt{\alpha }\left(1\;+\;k_{FF}\right)}{mh\tau }\left(\;h\;-\;2\tau \frac{1\;-\;k_{FF}}{1\;\;+k_{FF}}\;\right)\;\;>\;\left(\tau \;-\;h\right)\frac{\alpha }{mh^{2}\tau }\left(1\;-\;k_{FF}\right)\left(\;h\;-\;2\tau \frac{1\;-\;k_{FF}}{1\;+\;k_{FF}}\;\right),\\ \; & \frac{1+k_{FF}}{2m\tau }+\frac{\alpha k_{FF}+\sqrt{\alpha }\left(1+k_{FF}\right)}{mh\tau }\left(h-2\tau \frac{1-k_{FF}}{1+k_{FF}}\right)>\left(\tau -h\right)k_{p}, \end{array} \end{array}$$

where the second inequality is valid because the terms on the right-hand side are negative, while the fourth inequality holds due to the condition $h\left(1+k_{FF}\right)>2\tau \left(1-k_{FF}\right)$. Since the upper bound of $k_{d}$ in (46) exceeds $\left(\tau -h\right)k_{p}$, one can easily choose $k_{d}$ such that the condition (15b) is satisfied, i.e., $k_{d}>\left(\tau -h\right)k_{p}$. Consequently, the final selection of $k_{d}$ that satisfies both the individual vehicle stability and string stability conditions is

(48) $$\begin{array}{c} \begin{array}{cc} \; & \max\left\{\frac{1-k_{FF}}{mh}-\frac{\alpha (1-k_{FF})}{2mh\tau }\left(h-2\tau \frac{1-k_{FF}}{1+k_{FF}}\right),\;\left(\tau -h\right)k_{p}\right\}<k_{d}\\ \; & \;\;\;\;\;\;\;\;\;\;\;\;\leq \frac{1+k_{FF}}{2m\tau }+\frac{\alpha k_{FF}+\sqrt{\alpha }\left(1+k_{FF}\right)}{mh\tau }\left(h-2\tau \frac{1-k_{FF}}{1+k_{FF}}\right). \end{array} \end{array}$$

Second, let us consider the case where λ>1, and for this case, we will denote λ by β.

With

$$k_{p}=\frac{\beta }{mh^{2}\tau }\left(1-k_{FF}\right)\left(h-2\tau \frac{1-k_{FF}}{1+k_{FF}}\right),\;\mathrm{where}\;\beta >1,$$

the inequality in (26c), corresponding to condition (c1) in (24), is not satisfied. Hence, the derivative gain $k_{d}$ must be selected to satisfy condition (c2) in (24). The process described in (45) and (47) can be similarly applied by replacing α with β. In a manner analogous to (45), the following bounds are easily derived:

$$\begin{array}{c} \begin{array}{cc} \; & k_{d}>\frac{1+k_{FF}}{2m\tau }-\frac{\beta }{mh\tau }\left(h-2\tau \frac{1-k_{FF}}{1+k_{FF}}\right)\;\mathrm{and}\\ \; & \;\;\frac{1+k_{FF}}{2m\tau }+\frac{\beta k_{FF}-\sqrt{\beta }\left(1+k_{FF}\right)}{mh\tau }\left(h-2\tau \frac{1-k_{FF}}{1+k_{FF}}\right)\leq k_{d}\\ \; & \;\;\;\;\;\;\leq \frac{1+k_{FF}}{2m\tau }+\frac{\beta k_{FF}+\sqrt{\beta }\left(1+k_{FF}\right)}{mh\tau }\left(h-2\tau \frac{1-k_{FF}}{1+k_{FF}}\right), \end{array} \end{array}$$

$$\begin{array}{c} \begin{array}{cc} \; & \frac{1+k_{FF}}{2m\tau }+\frac{\beta k_{FF}-\sqrt{\beta }\left(1+k_{FF}\right)}{mh\tau }\left(h-2\tau \frac{1-k_{FF}}{1+k_{FF}}\right)\leq k_{d}\\ \; & \;\;\;\;\leq \frac{1+k_{FF}}{2m\tau }+\frac{\beta k_{FF}+\sqrt{\beta }\left(1+k_{FF}\right)}{mh\tau }\left(h-2\tau \frac{1-k_{FF}}{1+k_{FF}}\right),\; \end{array} \end{array}$$

where the inequality $\beta >\sqrt{\beta }$, valid for β>1, is applied to ensure that $-\beta <\beta k_{FF}-\sqrt{\beta }\left(1+k_{FF}\right)$. Furthermore, similar to (47), $k_{d}$ can always be chosen to satisfy the condition (15b) because we have

$$\begin{array}{c} \frac{1+k_{FF}}{2m\tau }+\frac{\beta k_{FF}+\sqrt{\beta }\left(1+k_{FF}\right)}{mh\tau }\left(h-2\tau \frac{1-k_{FF}}{1+k_{FF}}\right)>\left(\tau -h\right)k_{p}. \end{array}$$

Consequently, the final selection of $k_{d}$ to ensure both individual vehicle stability and string stability is given by

(49) $$\begin{array}{c} \begin{array}{cc} \; & \max\left\{\frac{1+k_{FF}}{2m\tau }+\frac{\beta k_{FF}-\sqrt{\beta }\left(1+k_{FF}\right)}{mh\tau }\left(h-2\tau \frac{1-k_{FF}}{1+k_{FF}}\right),\;\left(\tau -h\right)k_{p}\right\}<k_{d}\\ \; & \;\;\;\;\;\;\;\;\;\;\;\;\leq \frac{1+k_{FF}}{2m\tau }+\frac{\beta k_{FF}+\sqrt{\beta }\left(1+k_{FF}\right)}{mh\tau }\left(h-2\tau \frac{1-k_{FF}}{1+k_{FF}}\right). \end{array} \end{array}$$

## 5. Simulation Results

In this section, we examine the string stability of a five-vehicle platoon, including the leading vehicle. The platoon operates under a PD and feedforward-based CACC control scheme given in (8), and the controller gains are selected according to the design procedure in Algorithm 1. To examine the propagation of spacing errors, the desired acceleration of the leading vehicle in the driving scenario undergoes repeated acceleration and deceleration, with additional fluctuations defined in (50). These fluctuations introduce frequency components at 3 and 4.25 rad/s, which are particularly significant for analyzing resonance phenomena, as they may coincide with the peak frequencies of the transfer function in string instability cases, potentially amplifying disturbances and degrading string stability.

(50) $$\begin{array}{c} a_{\mathrm{fluc}}\left(t\right)=0.1\left(\sin(3t)+\sin(4.25t)\right). \end{array}$$

**Figure 4:**
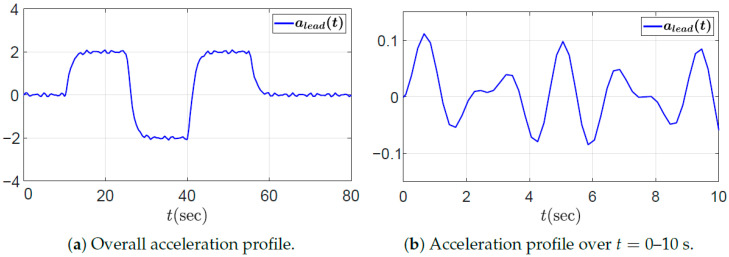
Acceleration profile of the leading vehicle $\left[m/s^{2}\right]$: (**a**) overall view; (**b**) zoomed-in view over the time interval t=0–10 s.

**Figure 5:**
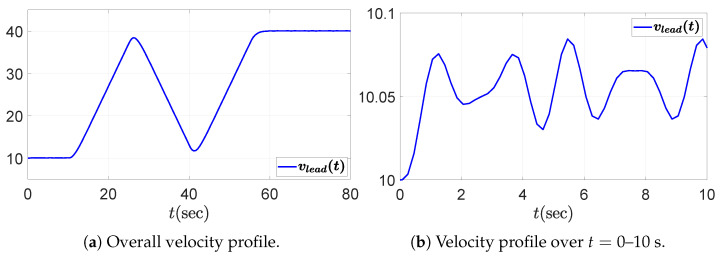
Velocity profile of the leading vehicle [m/s]: (**a**) overall view; (**b**) zoomed-in view over the time interval t=0–10 s.

If the platoon is not string stable, spacing errors will be amplified downstream. Conversely, in a string stable platoon, a properly well-designed controller will effectively attenuate these errors. The actual acceleration and velocity profiles of the leading vehicle, $a_{\mathrm{lead}}\left(t\right)$ and $v_{\mathrm{lead}}\left(t\right)$, are shown in Figure 4 and Figure 5, respectively, with zoomed-in views over the interval t=0–10s presented in the right panels. Notably, the zoomed-in acceleration profile in Figure 4b corresponds to the fluctuation signal of the desired acceleration defined in (50).

The simulation environment is completed by specifying the parameters of vehicle dynamics *m*, τ, and the time gap *h* as follows:

m=1,τ=0.5,h=0.2.

At time zero, the vehicles in the platoon are initially spaced 2 m apart, each traveling at a speed of 10 m/s with zero acceleration. To design a string stable controller and verify the feasible range of control gains, the feedforward gain $k_{FF}$ can be chosen first based on (39). Therefore, the possible range of $k_{FF}$ can be calculated as follows:

(51) $$\begin{array}{c} 0.667\leq k_{FF}<1. \end{array}$$

We choose $k_{FF}=0.8$ from the range (51).

In the first case study, the desired rise time is set to $t_{d,r}=3$ to determine the proportional gain $k_{p}$. In other words, the platoon system requires that each vehicle reach the desired spacing within 3 s when the preceding vehicle does not accelerate. According to (42), the proportional gain $k_{p}$ must satisfy

$$k_{p}>0.36.$$

We choose $k_{p}=0.7$, then (43) yields λ=0.7875≤1, which is the case of (48). Consequently, the allowable range for the derivative gain $k_{d}$ is determined by

(52) $$\begin{array}{c} 0.93<k_{d}\leq 3.780. \end{array}$$

**Figure 6:**
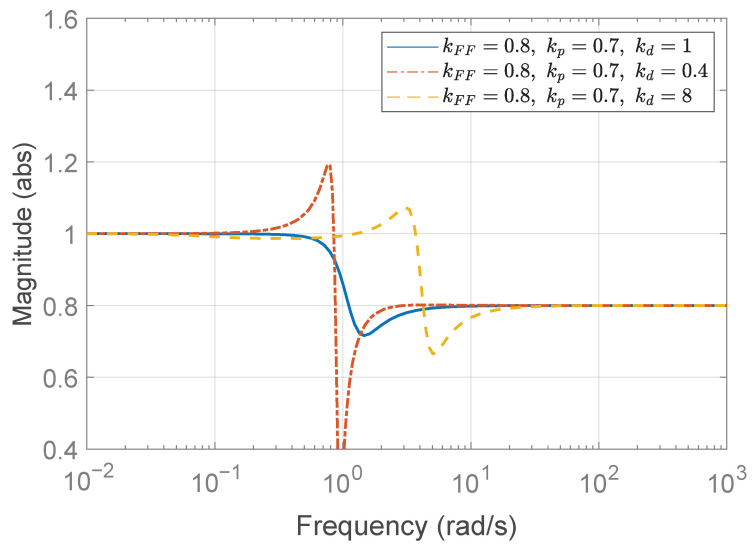
Bode magnitude plot of Γ(jω) for $k_{FF}=0.8\;\mathrm{and}\;k_{p}=0.7$ under different values of $k_{d}$: (**blue**) $k_{d}=1$; (**red**) $k_{d}=0.4$; (**yellow**) $k_{d}=8$.

Three representative values for the derivative gain are considered: (1) $k_{d}=1$, (2) $k_{d}=0.4$, and (3) $k_{d}=8$. Based on Theorem 1 and the corresponding range for $k_{d}$ derived in (52), it can be observed that $k_{d}=1$ satisfies the condition for string stability, whereas $k_{d}=0.4$ and $k_{d}=8$ do not. Figure 6 shows the Bode magnitude plot of Γ(jω), and it visualizes the violation of the string stability condition (14) for $k_{d}=0.4$ and $k_{d}=8$.

**Figure 7:**
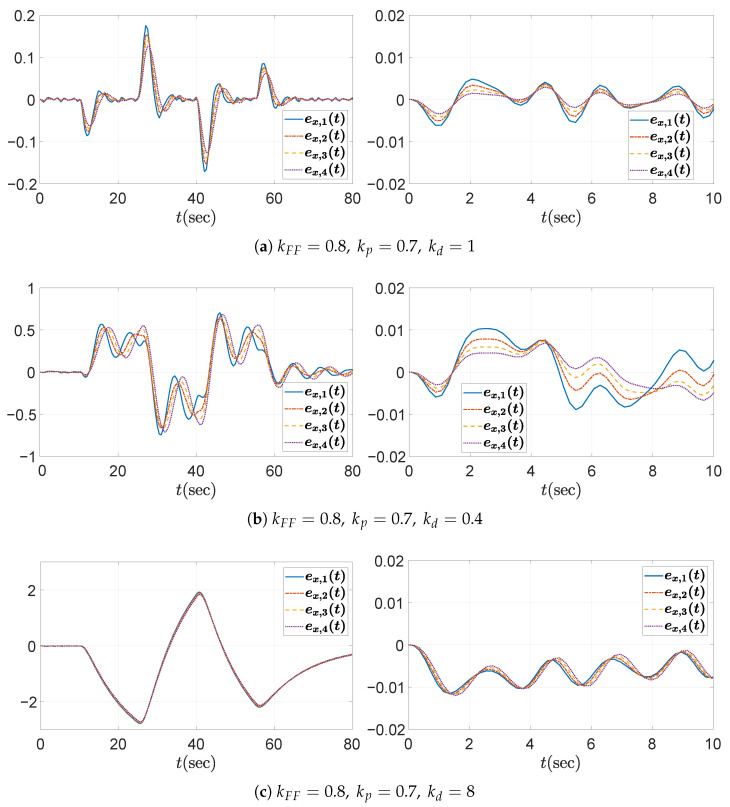
Headway distance error $e_{x,i}\left(t\right)\;\left[m\right]$ for $k_{FF}=0.8\;\;\mathrm{and}\;k_{p}=0.7$ under different values of $k_{d}$: (**left**) overall view; (**right**) zoomed-in view over the time interval t=0–10 s.

The temporal behaviors of the spacing errors $e_{x,i}\left(t\right)$ and their zoomed-in views are shown in Figure 7 for the three different $k_{d}$ cases. The left panel illustrates the spacing errors over the entire simulation duration (t=0–80 s), while the right panel provides a magnified view focusing on the interval t=0–10 s, during which the fluctuation signal defined in (50) and shown in Figure 4b is dominant. It is obvious from the overall view that $k_{d}=1$ ensures a string stable platoon, whereas $k_{d}=0.4$ does not. Moreover, during intervals of constant acceleration, ignoring minor fluctuations caused by (50), specifically t=15–25 s, 30–40 s, and 45–55 s, the spacing error converges to zero in steady state only when $k_{d}=1$. This is because, for $k_{d}=1$, the DC gain of the transfer function G(s) defined in (11) becomes zero, as expressed by

$$G\left(0\right)=\frac{\left(1-k_{FF}\right)-mhk_{d}}{mk_{p}}=0.$$

The case with $k_{d}=8$ is considered separately, as the platoon seems to exhibit string stability in the overall response shown in the left panel of Figure 7c. This observation may give the impression that increasing $k_{d}$ inherently promotes string stability in the platoon system; however, this is not generally guaranteed and must be examined with care. As illustrated in Figure 6, the yellow dashed line corresponding to the case of $k_{d}=8$ exceeds unity near 3 rad/s, indicating that string stability is not preserved for signals around this frequency. Although the overall response in Figure 7c (left panel) may suggest string stability for $k_{d}=8$, the zoomed-in view reveals otherwise. Due to the presence of a 3 rad/s component in the acceleration input $a_{\mathrm{fluc}}\left(t\right)$ defined in (50), string stability is not maintained, as this frequency corresponds to a peak in the transfer function magnitude exceeding unity. This highlights the limitation of relying solely on visual inspection of time domain responses. To robustly guarantee string stability, it is essential to follow the proposed design guideline.

Now, for the second case study, we take reduced rise time as $t_{d,r}=1.5$, which yields a possible range of $k_{p}$ given as

$$k_{p}>1.44$$

according to (42). With $k_{FF}=0.8$, as in the previous case, we choose $k_{p}=2.5$, then λ=2.8125>1 follows from (43), which is the case of (49). Accordingly, based on Algorithm 1, the feasible range of $k_{d}$ is calculated as follows:

(53) $$\begin{array}{cc} \; & 1.117<k_{d}\leq 6.483. \end{array}$$

**Figure 8:**
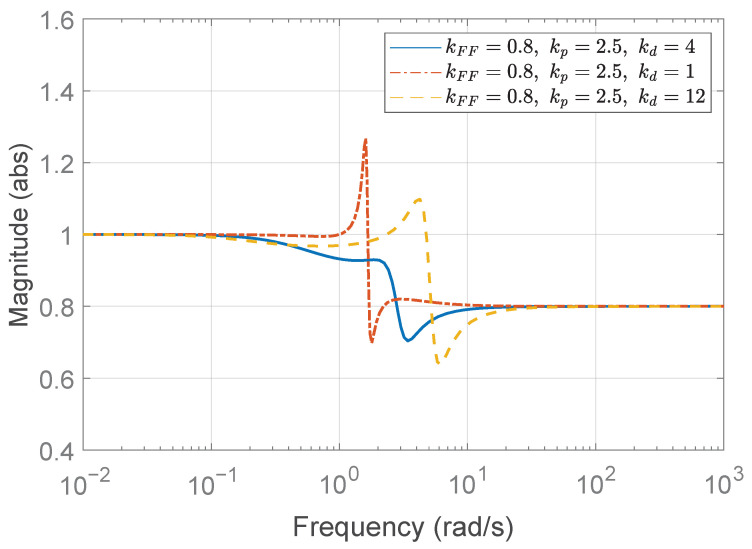
Bode magnitude plot of Γ(jω) for $k_{FF}=0.8\;\mathrm{and}\;k_{p}=2.5$ under different values of $k_{d}$: (**blue**) $k_{d}=4$; (**red**) $k_{d}=1$; (**yellow**) $k_{d}=12$.

Note that the allowable range of $k_{FF}$ remains unchanged from the previous case study, as Algorithm 1 indicates that the feedforward gain $k_{FF}$ is determined solely by τ and *h*, independently of the rise time specification $t_{d,r}$. Again, we consider the three different derivative gains: (1) $k_{d}=4$, (2) $k_{d}=1$, and (3) $k_{d}=12$. Figure 8 presents the Bode magnitude plots of Γ(jω) for the three derivative gains. As shown, only $k_{d}=4$, which lies within the stability range specified in (53), satisfies the string stability condition by maintaining the magnitude below unity.

**Figure 9:**
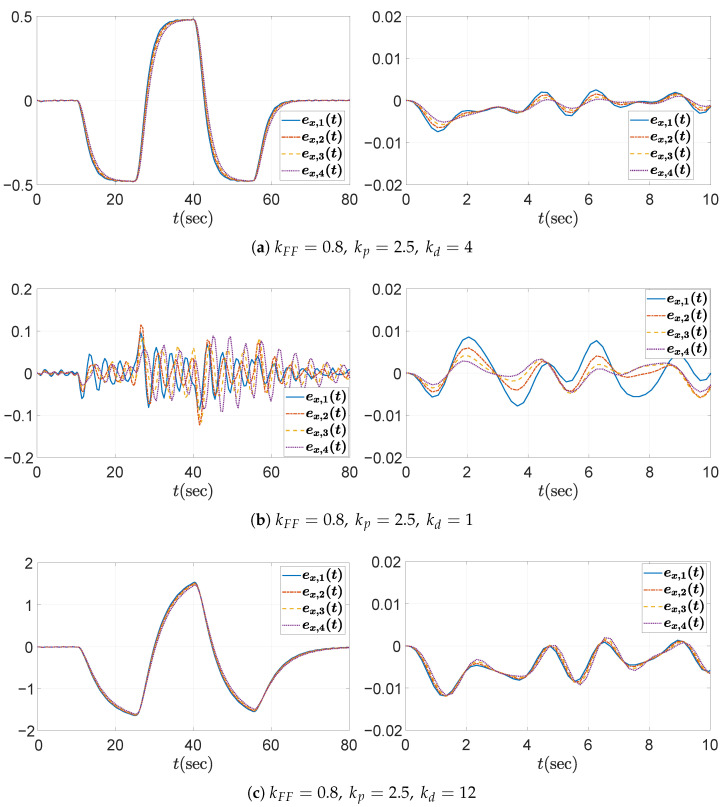
Headway distance error $e_{x,i}\left(t\right)\;\left[m\right]$ for $k_{FF}=0.8\;\;\mathrm{and}\;k_{p}=2.5$ under different values of $k_{d}$: (**left**) overall view; (**right**) zoomed-in view over the time interval t=0–10 s.

The corresponding temporal behavior of spacing errors is shown in Figure 9. Consistent with the first case study, the configuration with $k_{d}=4$ achieves string stability, while $k_{d}=1$ does not. Although the case with $k_{d}=12$ appears to maintain string stability in the overall view, the yellow dashed line in Figure 8 highlights the need to consider disturbances at peak frequencies—specifically, the 4.25 rad/s component in the fluctuation signal (50)—for practical applications. This is further illustrated in the zoomed-in view of the headway distance errors in Figure 9c (right panel), which clearly reveals string instability within the platoon. If external disturbances contain frequency components near the resonant peaks, string instability may be triggered, potentially leading to unsafe operating conditions for the platoon. Additionally, comparing Figure 9a,c reveals that the absolute magnitude of the spacing error is smaller in the string stable case with $k_{d}=4$ than in the string unstable case with $k_{d}=12$. Although the *y*-axis scale has been adjusted for clearer visualization, it is still evident that the string stable controller results in smaller errors. A final remark concerns the case of $k_{d}=1$. The spacing error of the individual vehicle converges to zero in steady state and remains smaller than that of the string stable case with $k_{d}=4$, particularly during the constant acceleration intervals t=15–25 s, 30–40 s, and 45–55 s. This behavior is attributed to the zero DC gain of the transfer function G(s) defined in (11), where $G\left(0\right)=\frac{\left(1-k_{FF}\right)-mhk_{d}}{mk_{p}}=0$. Nevertheless, the system is not string stable; thus, in a large platoon with many vehicles, the spacing errors can amplify toward the rear, potentially leading to unsafe driving conditions.

**Figure 10:**
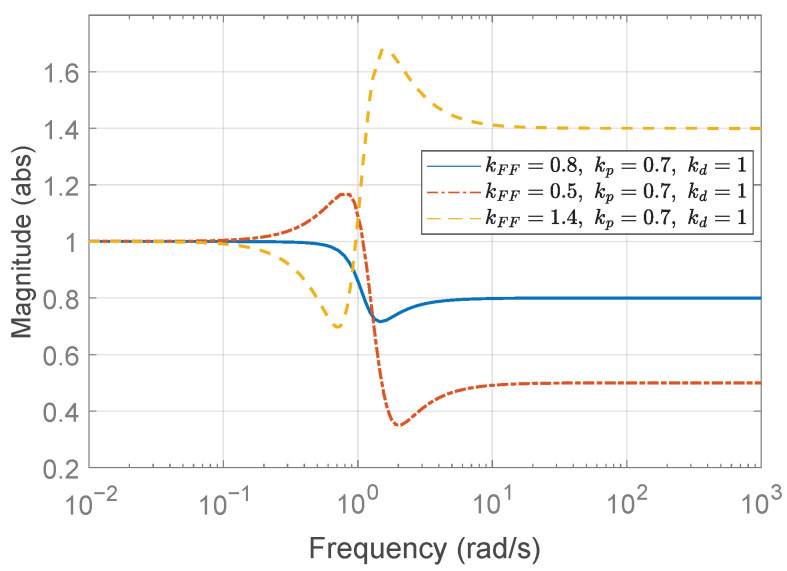
Bode magnitude plot of Γ(jω) for $k_{p}=0.7\;\mathrm{and}\;k_{d}=1.0$ under different values of $k_{FF}$: (**blue**) $k_{FF}=0.8$, (**red**) $k_{FF}=0.5$, (**yellow**) $k_{FF}=1.4$.

**Figure 11:**
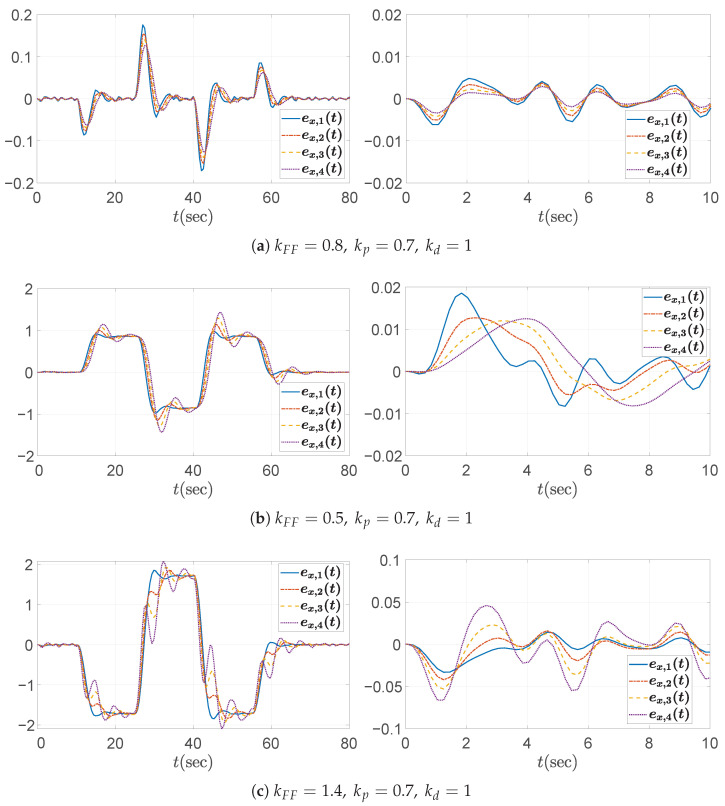
Headway distance error $e_{x,i}\left(t\right)\;\left[m\right]$ for $k_{p}=0.7\;\;\mathrm{and}\;k_{d}=1$ under different values of $k_{FF}$: (**left**) overall view; (**right**) zoomed-in view over the time interval t=0–10 s.

Lastly, the third case study investigates the effect of varying the feedforward gain $k_{FF}$. The proportional and derivative gains are fixed at $k_{p}=0.7$ and $k_{d}=1$, respectively, consistent with the string stable configuration from the first case study. Three feedforward gains are considered: (1) $k_{FF}=0.8$, (2) $k_{FF}=0.5$, and (3) $k_{FF}=1.4$. Among them, only $k_{FF}=0.8$ satisfies the condition given in (39), as explicitly derived in (51). As expected, the Bode magnitude plots in Figure 10 demonstrate that only the case with $k_{FF}=0.8$ satisfies the string stability criterion, maintaining a magnitude below unity, whereas the other cases clearly violate this condition. The corresponding spacing error responses, illustrated in Figure 11, further emphasize the critical importance of proper feedforward gain selection, confirming that only $k_{FF}=0.8$ maintains string stability.

**Figure 12:**
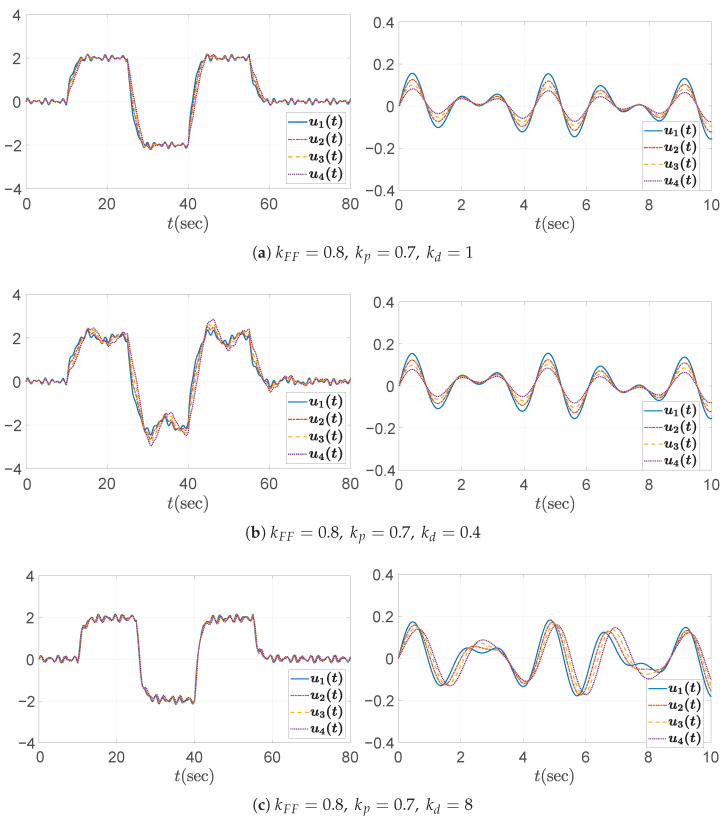
Control input $u_{i}\left(t\right)\;=\;a_{d,i}\left(t\right)\;\left[m/s^{2}\right]$ for $k_{FF}\;=0.8\;\;\mathrm{and}\;k_{p}\;=0.7$ under different values of $k_{d}$: (**left**) overall view; (**right**) zoomed-in view over the time interval t=0–10 s.

**Figure 13:**
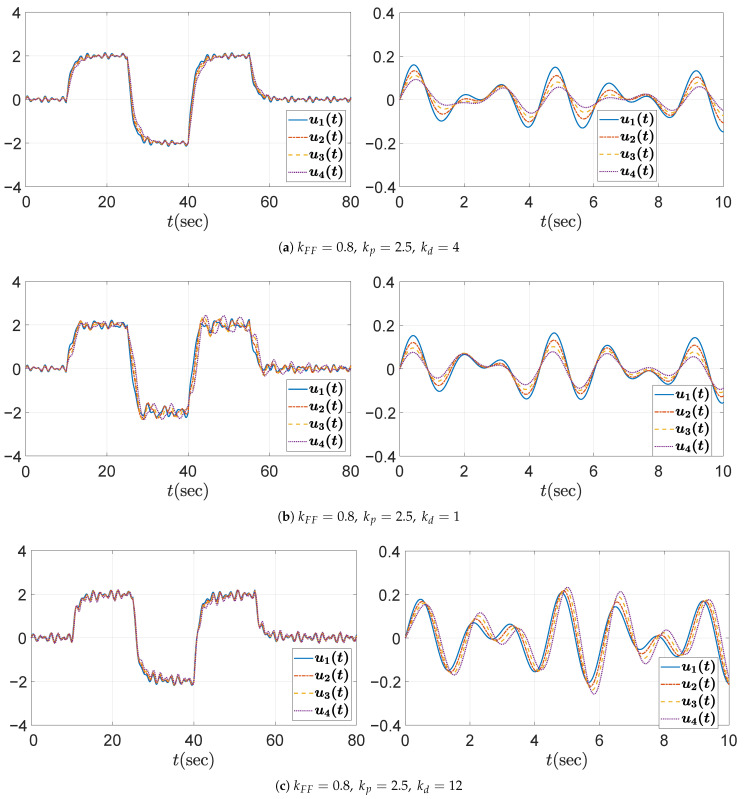
Control input $u_{i}\left(t\right)\;=\;a_{d,i}\left(t\right)\;\left[m/s^{2}\right]$ for $k_{FF}\;=0.8\;\;\mathrm{and}\;k_{p}\;=2.5$ under different values of $k_{d}$: (**left**) overall view; (**right**) zoomed-in view over the time interval t=0–10 s.

**Figure 14:**
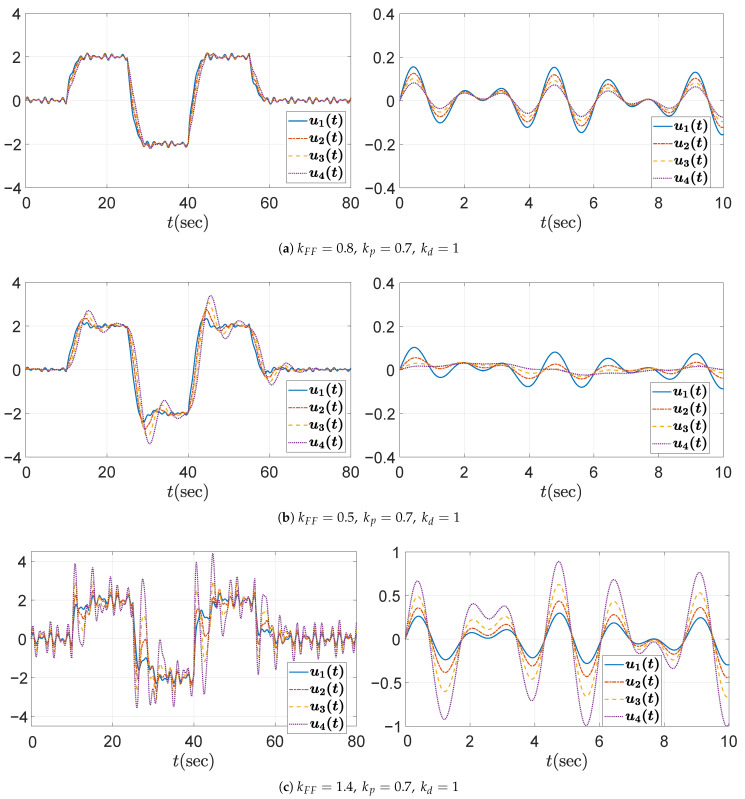
Control input $u_{i}\left(t\right)\;=\;a_{d,i}\left(t\right)\;\left[m/s^{2}\right]$ for $k_{p}=0.7\;\;\mathrm{and}\;k_{d}=1$ under different values of $k_{FF}$: (**left**) overall view; (**right**) zoomed-in view over the time interval t=0–10 s.

As mentioned in Section 3.1 and Equation (13), it was confirmed that the error string stability function H(s) and the input string stability function Γ(s) are identical in a homogeneous platoon. Therefore, the string stability property should be reflected not only in the distance errors $e_{x,i}$, but also in the control inputs $u_{i}=a_{d,i}$. Figure 12, Figure 13 and Figure 14 show that the control inputs in the three simulation cases are also string stable, consistent with the string stable headway distance errors presented in Figure 7, Figure 9, and Figure 11. Moreover, the behavioral trend of the control inputs exhibits similar patterns to those of the distance errors, further confirming the equivalence between input and error string stability in a homogeneous platoon. From the figures, we observe that only string stable controllers show the relation $∥u_{i}∥\leq ∥u_{i-1}∥$. Based on these observations, we conclude that the limit of the control inputs or input constraints is unlikely to pose a significant concern, provided that the design guideline in Algorithm 1 is properly followed and the desired acceleration input of the leading vehicle remains within reasonable bounds.

All tested combinations of control gains and their corresponding stability evaluations are summarized in Table 3, where the symbol ◯ indicates that a specified condition is satisfied, and × indicates otherwise. The simulation results confirm that the ranges of control gains determined by Algorithm 1 are consistent with the theoretical requirements for ensuring both string stability and individual vehicle stability. Table 3 provides a consolidated overview of the feasibility assessments for the eight parameter sets considered in the three case studies.

## 6. Conclusions

This paper presents a systematic framework for selecting control gains in CACC-based platoon systems, aiming to ensure both individual vehicle stability and string stability for a homogeneous platoon with identical longitudinal dynamics. The control strategy is grounded in a PD and feedforward controller, where the proportional gain $k_{p}$, derivative gain $k_{d}$, and feedforward gain $k_{FF}$ must be carefully selected to meet the required stability conditions. A key contribution of this study lies in demonstrating that string stability can be effectively achieved in CACC systems through the use of simple and practical PD control strategies combined with static feedforward compensation. We begin with a detailed examination of the necessary and sufficient conditions for both individual vehicle stability and string stability, and use this analysis to formulate practical design guidelines for selecting appropriate control gains. The proposed methodology also incorporates communication delays, which are critical in real-world implementations, and addresses scenarios where the actual acceleration of the preceding vehicle is used in the feedforward path in place of the desired acceleration. Overall, the resulting framework provides a comprehensive and practical resource for engineers deploying CACC in real-world vehicular platoons, effectively bridging theoretical analysis with implementation concerns.

Furthermore, a notable limitation of this study is the assumption of homogeneous longitudinal dynamics across all vehicles in the platoon, neglecting any model uncertainties or inter-vehicle variations. While this assumption simplifies the analysis and allows for straightforward controller design, it does not reflect the diversity commonly encountered in real-world vehicular systems, where variations and uncertainties in vehicle mass, actuator dynamics, engine response, and braking performance are inevitable and must be considered for realistic deployment. Such heterogeneity and uncertainty introduce significant challenges in maintaining both individual vehicle stability and string stability, as controllers tuned for a nominal vehicle model may experience degraded performance or even instability when applied to diverse real-world vehicles. To address this issue, future research should focus on extending the proposed gain selection framework to uncertain heterogeneous vehicle platoons. This necessitates the development of robust control strategies capable of accommodating vehicle variability and uncertainty while maintaining consistent performance across diverse dynamic behaviors. A promising approach is the incorporation of disturbance observers (DOBs) [41,42,43,44], which estimate and compensate for model uncertainties and external disturbances in real time. By reconstructing the discrepancy between the actual and nominal vehicle dynamics, DOB-based controllers allow each vehicle to operate as if it follows a unified nominal model. This not only improves robustness but also supports scalable deployment of CACC systems across uncertain heterogeneous vehicle fleets. Successful applications of DOB-based control techniques in addressing model uncertainty and vehicle heterogeneity, as well as in enhancing string stability, have been demonstrated in previous studies [45,46,47]. Integrating such techniques into the proposed gain tuning framework, supported by rigorous theoretical analysis, could significantly improve its robustness and practical applicability in mixed traffic scenarios.

## Figures and Tables

**Table 1:** Conditions for individual vehicle stability and string stability.

Property	Individual Vehicle Stability	String Stability
Transfer Function	G(s) in (11)	Γ(s) in (10)
Original Condition	G(s) is stable (i.e., D(s) in (12) is Hurwitz)	${∥\Gamma \left(j\omega \right)∥}_{\infty }\leq 1$ (i.e., (14))
Condition on Parameter	(15a) and (15b)	(c1) or (c2) in (24)

**Table 2:** Routh array of D(s).

$s^{3}\;$	τ	$\;m(hk_{p}+k_{d})$
$s^{2}\;$	1	$\;mk_{p}$
$s^{1}\;$	$\;m\left(hk_{p}+k_{d}\right)-m\tau k_{p}\;$	0
$s^{0}\;$	$\;mk_{p}$	

**Table 3:** Feasibility check of individual vehicle stability and string stability under given parameters.

Model				Control Gains				Parameter Conditions				Stability Conditions			
	m	τ	h		$k_{\mathrm{FF}}$	$k_{p}$	$k_{d}$		$k_{\mathrm{FF}}$ (39)	$k_{p}$ (15a)	$k_{d}$ (48)/(49)		**Individual** (15)	**String** (24)	
					0.5				×	◯	N/A		◯	×	
					1.4		1		×	◯	N/A		◯	×	
						0.7			◯	◯	◯-(48)		◯	◯-(c1)	
	1	0.5	0.2				0.4		◯	◯	×		◯	×	
					0.8		8		◯	◯	×		◯	×	
							4		◯	◯	◯-(49)		◯	◯-(c2)	
						2.5	1		◯	◯	×		◯	×	
							12		◯	◯	×		◯	×	

## Data Availability

Data are contained in the article.
